# Abnormal basement membrane results in increased keratinocyte-derived periostin expression in psoriasis similar to wound healing

**DOI:** 10.1038/s41598-023-43396-0

**Published:** 2023-09-29

**Authors:** Lili Borbála Flink, Ameneh Ghaffarinia, Benjamin Tamás Papp, Ákos Varga, András István Vigh, Dániel László Vidács, Róbert Kui, Lajos Kemény, Zsuzsanna Bata-Csörgő, Renáta Bozó

**Affiliations:** 1 Department of Dermatology and Allergology, Albert Szent-Györgyi Medical School, University of Szeged, Korányi Street 6, Szeged, 6720 Hungary; 2 HCEMM-USZ Skin Research Group, University of Szeged, Szeged, 6720 Hungary; 3 HUN-REN–SZTE Dermatological Research Group, Hungarian Research Network, Szeged, 6720 Hungary

**Keywords:** Skin diseases, Autoimmunity, Skin models, Experimental models of disease

## Abstract

The psoriatic skin resembles wound healing, and it shows abnormalities at the basement membrane (BM), also in the non-lesional skin. Fibroblast-derived dermal periostin has well-known functions in wound healing and Th2-mediated diseases, such as atopic dermatitis. Here we show that serum periostin level was elevated in psoriatic patients, remarkably in the systemically treated ones. Obvious periostin positivity was detected in basal keratinocytes of the non-lesional, lesional, and previously-lesional psoriatic vs. healthy skin. Ex vivo skin models were generated to examine how different skin injuries affect periostin expression during wound healing. Our newly developed cultured salt-split model demonstrated that BM-injury induced periostin expression in basal keratinocytes, and periostin levels in the supernatant were also increased upon healing. In wound healing models, β1-integrin expression was similarly induced. β1-integrin blocking caused reduced periostin expression in in vitro scratch assay, indicating that β1-integrin can mediate periostin production. In contrast to atopic dermatitis, psoriatic basal keratinocytes are in an activated state and show a stable wound healing-like phenotype with the overexpression of periostin. This abnormal BM-induced wound healing as a potential compensatory mechanism can be initiated already in the non-lesional skin present in the lesion and keratinocytes can remain activated in the healed skin.

## Introduction

Chronic plaque-type psoriasis is a multifactorial, mainly Th1 and Th17 pathway-mediated inflammatory skin disease, which is the most frequent type of psoriasis^1^ with characteristic red, scaly patches. It is characterized by epidermal hyperplasia, massive infiltration of immune cells and altered basement membrane (BM) composition with only partially understood pathomechanism. Numerous data indicate that alterations of the dermal–epidermal junction region and BM zone are already present in the phenotypically healthy-looking, non-lesional psoriatic skin^2–5^. It is well established that psoriasis is also associated with other diseases, most often with psoriatic arthritis^6,7^, but emerging studies suggest an association with obesity, mental disorders, cardiovascular and metabolic diseases^1,8,9^, although it needs to be further investigated whether psoriasis itself is a risk factor for these diseases^1^. Genes and environmental factors play crucial roles in the development of psoriasis, and disease manifestation requires both interactions^1^.

Recent therapies are able to induce complete resolution of the symptoms, but if treatment is suspended, symptoms may occur again very often at the same body sites, indicating that in resolved lesions a molecular scar remains^10^, and epigenetic changes detected in epidermal keratinocytes of resolved skin may be responsible for the disease residual transcriptomic profile found in the same regions^11^.

Periostin is an extracellular matrix component, in the skin it is mainly located in the papillary dermis and at the dermal–epidermal junction. It is well established that periostin plays a vital role in wound healing by maintaining tissue structure, inducing proliferation and differentiation of epithelial cells, and contributing to fibroblast activation and fibroblasts myofibroblasts transformation after transforming growth factor beta activation^12,13^.

The role of periostin has been widely investigated in atopic dermatitis, a skin disease with very different immunopathology compared to psoriasis. Periostin has been shown to play a role in Th2 pathway-mediated inflammatory diseases, such as atopic dermatitis, where interleukin-4 and interleukin-13 cytokines have been reported to activate periostin production in fibroblasts. Periostin was shown to be elevated not only in the inflamed dermis but also in the serum of atopic dermatitis patients and its level correlated with disease severity suggesting that periostin is an accelerator of atopic dermatitis progress^14–16^.

The role of periostin in wound healing and other inflammatory skin diseases, such as atopic dermatitis, is relatively well-known^13,14,16,17^. However, its potential role in the pathogenesis of psoriasis remains undetermined.

Here we show that serum and basal keratinocyte periostin expression is elevated in psoriasis. Our different ex vivo skin wound healing models, particularly our BM injury model, revealed increased periostin expression in basal keratinocytes and increased presence of periostin in the supernatant upon healing. We also found that besides periostin, β1-integrin expression was similarly elevated in basal keratinocytes in our models and the increased periostin expression was likely mediated by β1-integrin. These results indicate the role of periostin in the wound healing phenotype of psoriatic basal keratinocytes, which may be the result of BM abnormalities found in psoriatic skin.

## Results

### Serum periostin is increased in psoriatic patients

**Figure 1:**
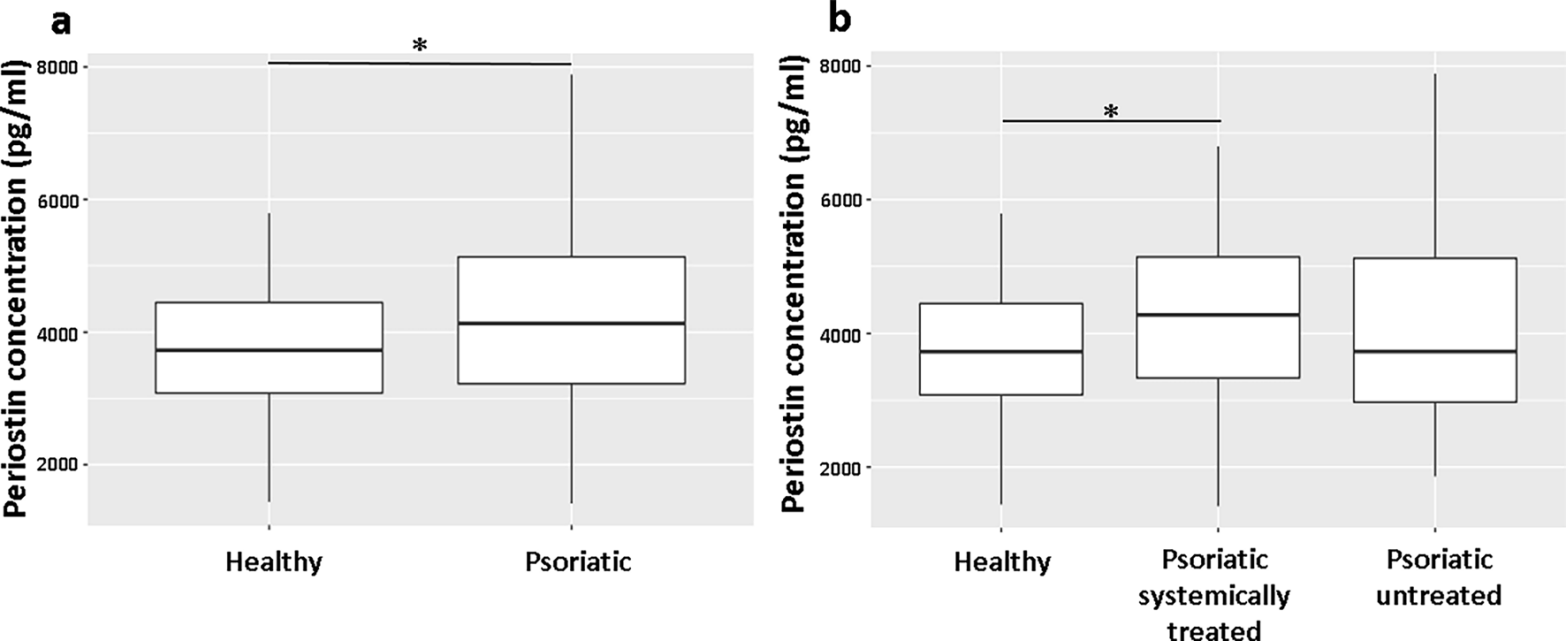
Serum periostin level is elevated in psoriatic patients, especially in patients with systemic therapy. (**a**) Significantly elevated serum periostin levels were measured using sandwich ELISA assay in psoriatic patients (n = 105) compared to healthy individuals (n = 49). *P* values are determined by two-sided two-sample *t*-tests. (**b**) Periostin levels were measured in untreated (n = 41), systemically treated psoriatic patients (n = 64), and healthy individuals (n = 49). FDR-adjusted *P* values are calculated by the Kruskal–Wallis test followed by the pairwise Wilcoxon test. Median serum periostin values are indicated by horizontal bars, the top and bottom of the box represent the lower and upper quartiles, and vertical lines show the outliners. *P < 0.05 versus healthy controls.

Among serum inflammatory markers (VEGF, survivin, uPar, fibronectin, data not shown) we found that periostin was significantly elevated in psoriatic patients, which is in agreement with previous data^14^ (Fig. 1a). Interestingly, among all patients, the systemically treated group showed the highest elevation in periostin serum level (Fig. 1b). We did not observe significant differences between male and female patients (Supplementary Fig. S1a) or between younger and older patients (Supplementary Fig. S1b), and the Body Mass Index (BMI) did not influence on the measured periostin level (Supplementary Fig. S1c).

As opposed to atopic dermatitis, in which periostin serum levels are closely related to the severity and activity of the disease^14^, in psoriasis serum periostin levels did not correlate with the severity of the disease (Supplementary Fig. S2a), even when we looked separately in groups of 15 and > 15 Psoriasis Area Severity Index (PASI) score patients (data not shown). We compared serum periostin levels in patients on biological vs. other systemic therapies, and no significant difference was found (Supplementary Fig. S2b). Periostin mRNA levels in healthy, non-lesional, and lesional psoriatic skin were also analyzed to determine the periostin expression in the skin using data from the publicly available GEO Profile dataset. We found significantly decreased periostin mRNA expression in lesional skin compared to non-lesional and healthy skin samples (Supplementary Fig. S3).

### Periostin expression is elevated in basal keratinocytes but not in the dermis of psoriatic skin

**Figure 2:**
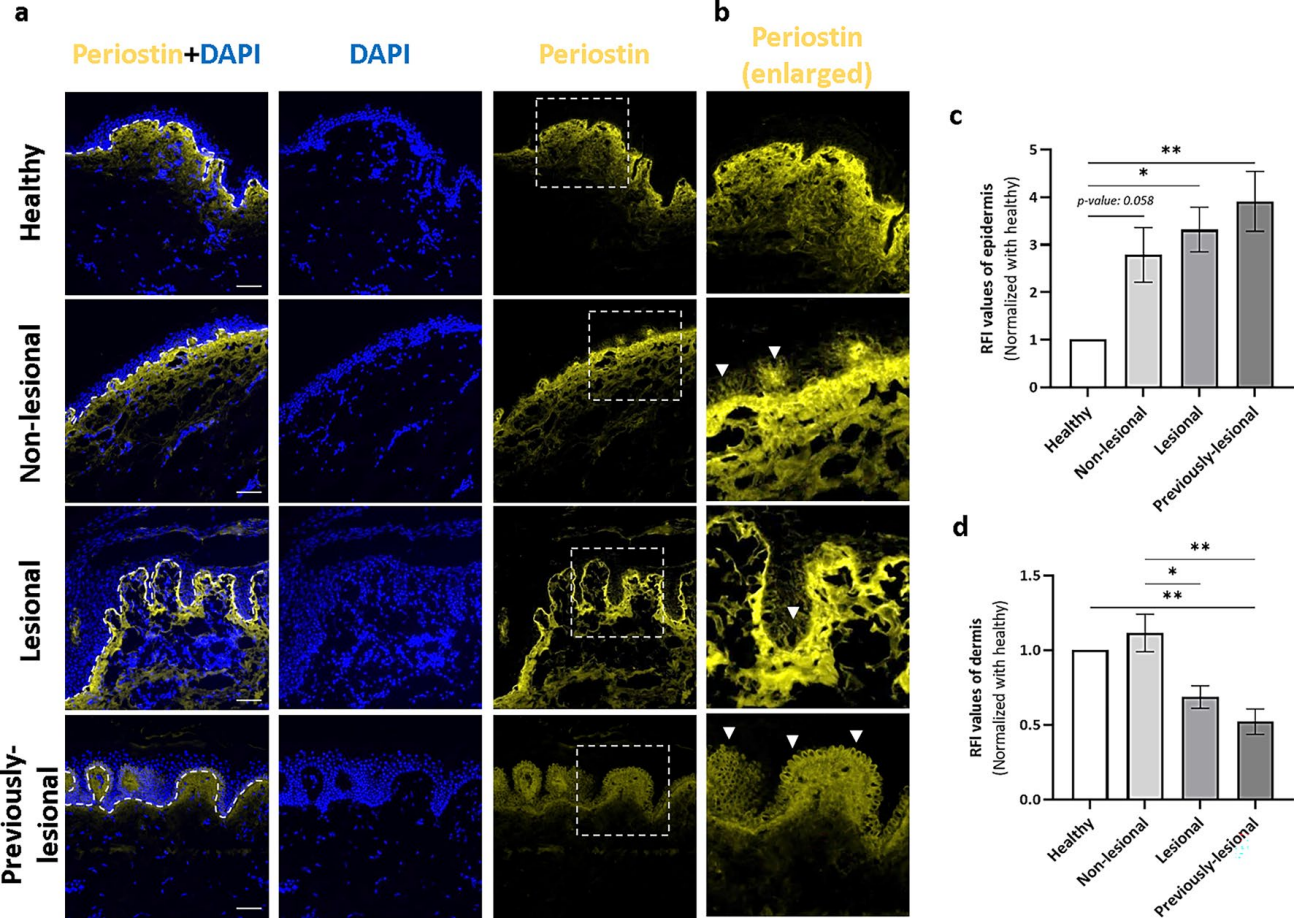
Periostin expression is reduced in the lesional and previously-lesional dermis and increased in the non-lesional, lesional, and previously-lesional psoriatic epidermis. (**a**) Immunofluorescence staining of periostin in healthy, psoriatic non-lesional, lesional, and previously-lesional skin. Representative pictures from 4 independent donors are shown. Dotted lines highlight the border of the epidermis and dermis, dotted rectangles indicate the enlarged regions, magnification: 20x. Bar = 50 µm. (**b**) Periostin is present in the layer of basal keratinocytes in the non-lesional, lesional, and previously-lesional skin. Arrows indicate the positive cell layer. (**c**) Relative fluorescence intensity (RFI) measurement of periostin in the dermis of the healthy, non-lesional, lesional, and previously-lesional skin. (**d**) RFI measurement of the healthy, non-lesional, lesional, and previously-lesional epidermis. Data represent the mean ± SEM (n = 4). RFI values were tested for significance by using one-way ANOVA followed by Tukey’s post-hoc test. *P < 0.05 and **P < 0.01 were considered significant.

In normal skin, periostin is known to be localized at the papillary dermis^15^. Investigation of periostin expression in the healthy, non-lesional, and lesional skin of untreated patients as well as in the previously-lesional, healed psoriatic skin by immunofluorescence labeling revealed decreased protein levels in the dermis of lesional skin, but not in the non-lesional skin compared to healthy skin (Fig. 2a). The lowest dermal periostin expression was observed in the previously-lesional skin (Fig. 2d). At the same time, immunofluorescence staining also revealed a statistically significant increase in periostin expression of basal keratinocytes in the lesional and previously-lesional healed epidermis, and it was nearly significant in the non-lesional skin in contrast to healthy skin (Fig. 2b,c) based on relative fluorescence intensity (RFI). With western blot analysis, we found significantly decreased periostin levels in lesional and previously-lesional protein extracts from whole skin punch biopsies versus healthy skin (Supplementary Fig. S4a and S4b). In previously-lesional skin, as opposed to decreased periostin at the dermal–epidermal junction, basal keratinocytes showed the highest expression (Fig. 2b). Western blot analysis revealed that periostin expression of keratinocytes derived from previously-lesional psoriatic skin was increased compared to healthy cells (Supplementary Fig. S4c and S4d), suggesting a correlation with the immunofluorescence staining results. Periostin is known to be expressed by both keratinocytes and fibroblasts to different molecular effects^17^. We examined how healthy cultured fibroblasts and normal human epidermal keratinocytes express periostin and we also found that both cultured cell types can produce periostin, however, the monomeric form of periostin was more characteristic of fibroblasts (Supplementary Fig. S5).

### Periostin expression is more intense in ex vivo wound healing- and cultured salt-split models in contrast to tape-stripping models

**Figure 3:**
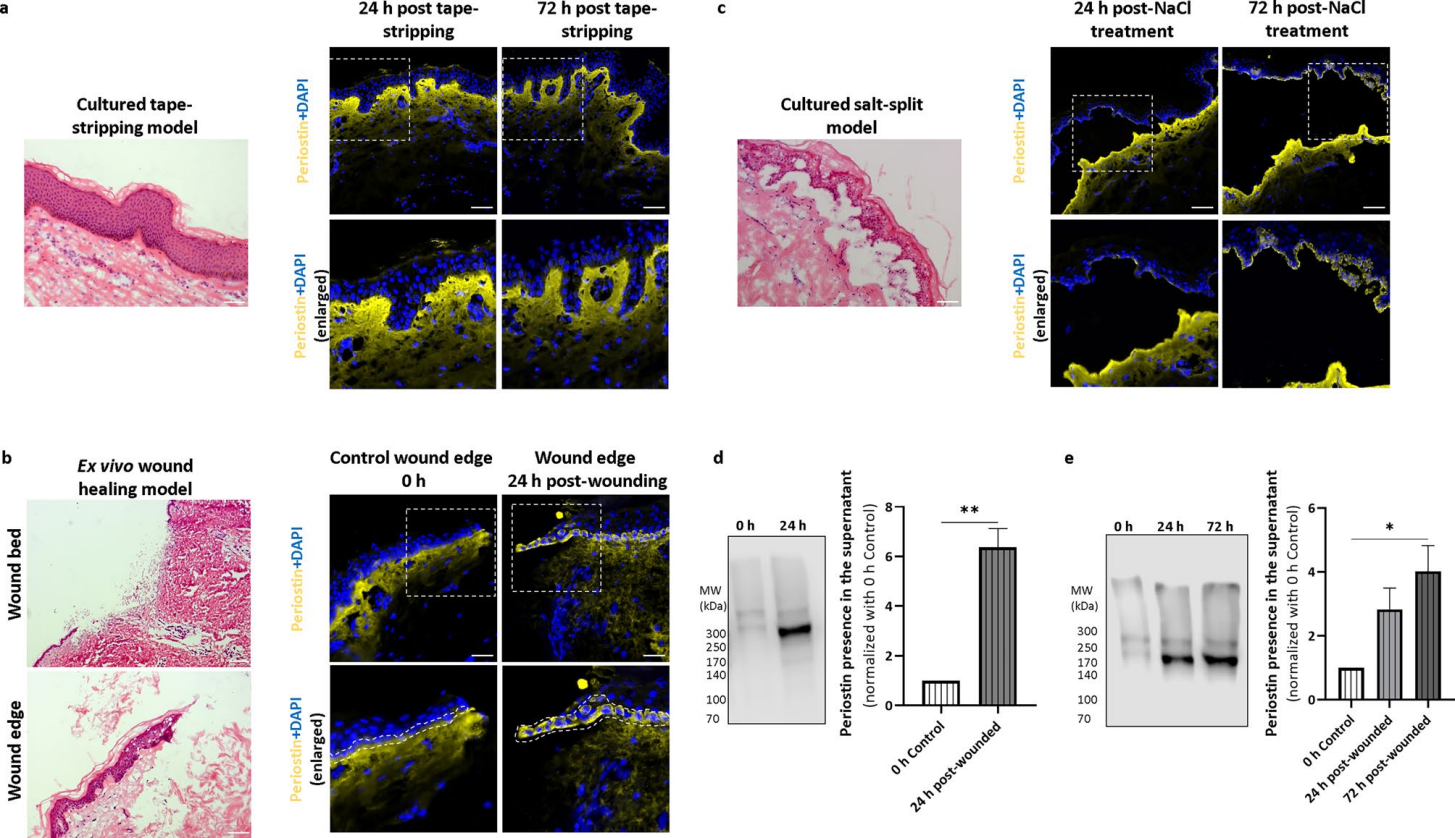
Periostin expression of basal keratinocytes and the presence of periostin in the supernatant are increased in cutting-type wound healing and cultured salt-split models but not in tape-stripping models. Representative images of hematoxylin–eosin and periostin immunofluorescence staining (**a**) in tape-stripping models at 24 and 72 h post-treatment (n = 3), (**b**) in cutting-type ex vivo skin wound healing models at 0 and 24 h post wounding (n = 5, basal keratinocytes are highlighted by dotted lines, magnification in the hematoxylin–eosin stained wound bed: 2,5x, bar = 250 µm), and (**c**) in cultured salt-split basement membrane injury models at 24 and 72 h post-injury (n = 5). Dotted rectangles indicate all the enlarged regions, magnification: × 20. Bar = 50 µm. The graphs show mean ± SEM (n = 3) of the periostin presence in the supernatant of the (**d**) ex vivo wound healing at 0 and 24 h, and (**e**) cultured salt-split models at 0, 24 and 72 h using western blot analysis. Data were normalized to 0 h control samples, **P < 0.01, determined by one-tailed two-sample *t*-test; *P < 0.05, determined by one-way ANOVA followed by Tukey’s posthoc test**.** Band intensities of periostin were quantitated with Image Studio software (LI-COR Biosciences, Lincoln, NE). Representative blots are shown.

In order to examine the effect of different skin injuries on periostin expression, we used various wounding types using ex vivo skin: tape-stripping, to model barrier disruption characteristic for atopic dermatitis; cutting through the tissue as a classical 3D ex vivo wound healing model and we newly developed a cultured salt-split model, where only the BM was wounded. In the tape-stripping model, there was no obvious periostin expression in basal keratinocytes at 24- and 72 h (Fig. 3a). In the cutting model, we observed a prominent periostin expression in basal keratinocytes at the wound edges after 24 h (Fig. 3b).

Cultured salt-split samples, the models for BM injury, revealed increased periostin expression in basal keratinocytes after 72 h compared to 24 h’ samples post-injury (Fig. 3c). Since we observed increased expression of periostin by basal keratinocytes, we collected supernatant at 0 and 24 h post-wounding from the cutting model as well as from the cultured salt-split model at 0, 24, and 72 h post-wounding. We found similarly increased elevated periostin levels in the supernatant to what we observed in basal keratinocytes at 24 and 72 h upon cutting or salt-split. (Fig. 3d,e).

### Parallel with increased periostin, β1-integrin expression is also increased in basal keratinocytes in ex vivo cultured and salt split wound healing models

**Figure 4:**
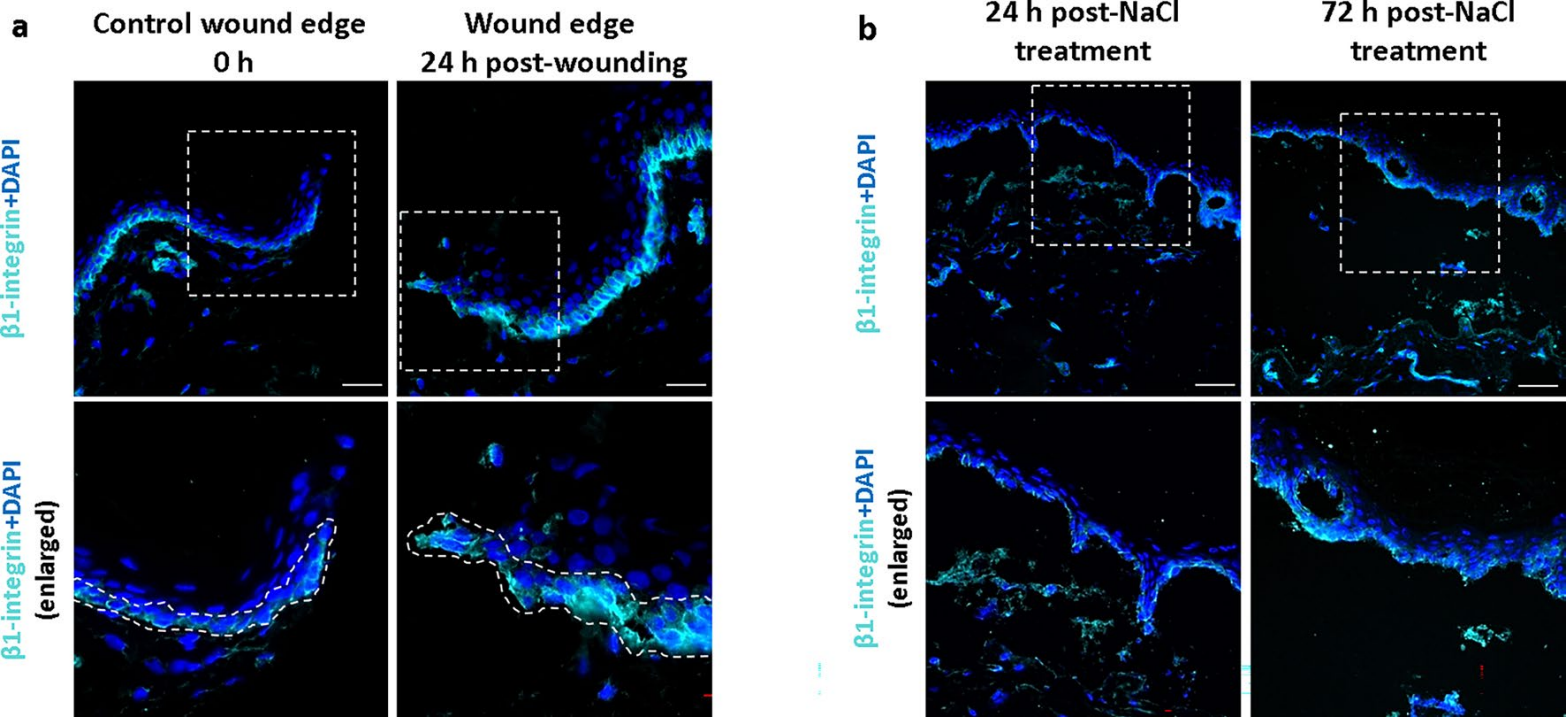
β1-integrin expression of basal keratinocytes is increased in ex vivo wound healing- and cultured salt-split models. Immunofluorescence labeling of β1-integrin in (**a**) cutting-type ex vivo wound healing models at 0 and 24 h (n = 5, basal keratinocytes are highlighted by dotted lines), and (**b**) in cultured salt-split models (n = 3) at 24 and 72 h. Dotted rectangles indicate all the enlarged regions, magnification: × 20. Bar = 50 µm.

Our previous data suggested a crucial role of β1-integrin in the stabilization of the epidermis upon BM disruption in the psoriatic non-lesional skin^18^. Examining whether β1-integrin on basal keratinocytes can perceive injuries and potentially contribute to the BM-injury induced increased expression of periostin, immunofluorescence staining was performed on the cultured cutting-type and salt-split ex vivo models. Similar to periostin, we also detected increased β1-integrin expression by basal keratinocytes at 24 h post-wounding in the cutting-type as well as in the cultured salt-split models after 72 h compared to 24 h (Fig. 4a,b).

### Periostin expression is reduced upon blocking β1-integrin in normal human epidermal keratinocytes

**Figure 5:**
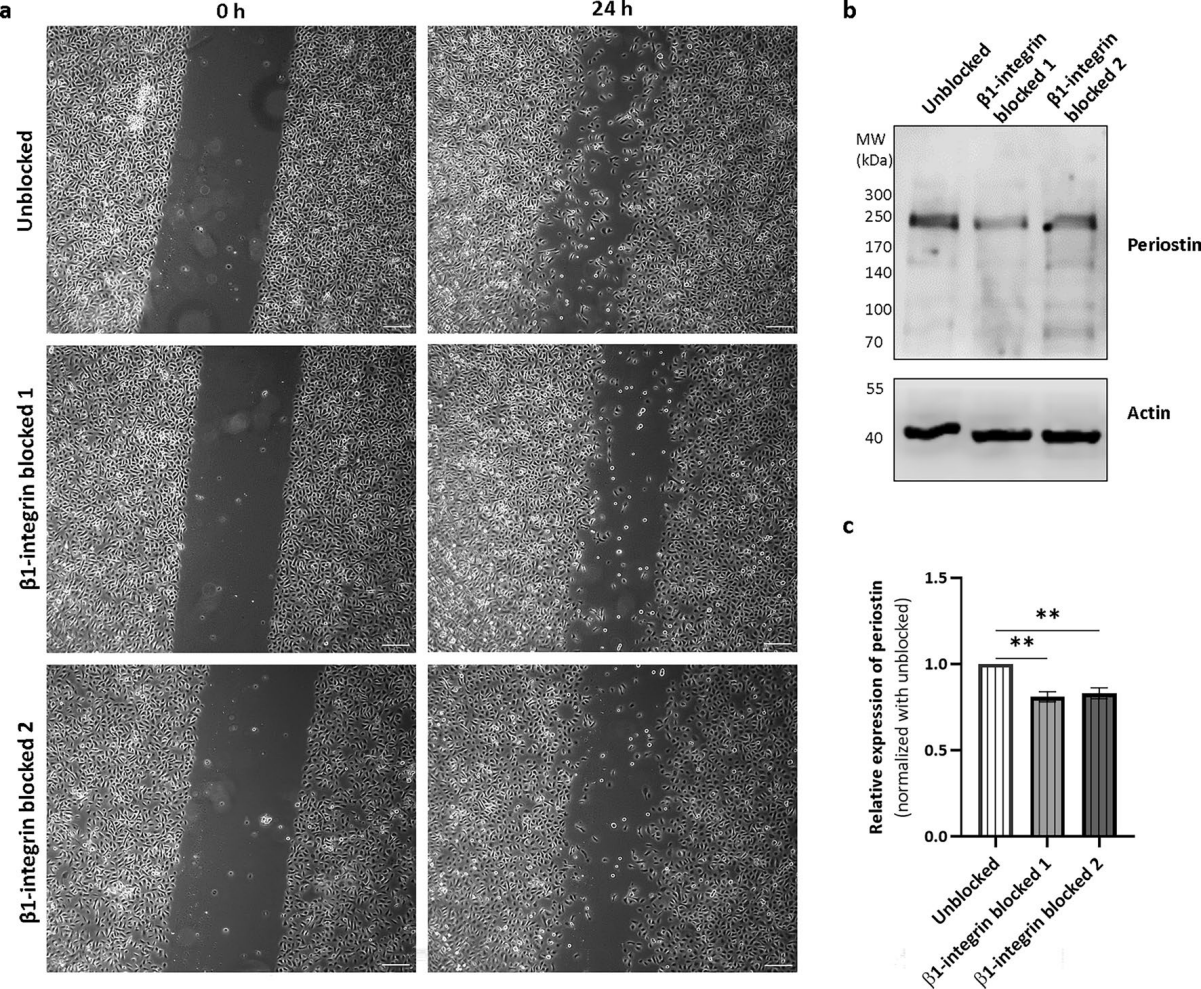
β1-integrin blocking on normal human epidermal keratinocytes resulted in delayed wound healing and reduced periostin expression. (**a**) Representative images of in vitro scratch assay on primary normal human keratinocytes combined with β1-integrin blocking with two different antibodies at 0 and 24 h post wounding (n = 3, magnification: × 5. Bar = 200 µm). (**b**) Representative blot of periostin in keratinocyte lysates from in vitro scratch assays (n = 3). (**c**) The graph shows the mean ± SEM (n = 3) of the periostin lysates. Actin was used as a loading control. Band intensities of periostin were quantitated with Image Studio software (LI-COR Biosciences, Lincoln, NE) and presented as fold changes normalized to actin. **P < 0.01, determined by one-way ANOVA followed by Tukey post hoc test.

To investigate whether β1-integrin could mediate the expression of periostin, β1-integrin-blocking was applied in in vitro scratch wound healing assay using normal human keratinocytes. Blocking β1-integrin resulted in delayed closure of the wounds compared to unblocked normal keratinocytes (Fig. 5a). Western blot analysis revealed that periostin production was reduced in keratinocytes due to blocking β1-integrin compared to unblocked control (Fig. 5b,c). These results indicate that β1-integrin is needed for proper wound healing and it contributes to the induction of periostin.

## Discussion

Increasing evidence suggests that psoriasis not only affects the skin but can be considered as a systemic inflammatory disease with abnormalities present in the circulation^19^. Periostin is involved in different inflammatory conditions such as asthma, atherosclerosis, rheumatoid arthritis, and other skin diseases, such as atopic dermatitis^14,20–23^. Increased serum periostin is detected in patients with atopic dermatitis and psoriasis, but its level is the highest in atopic dermatitis and correlates with disease severity. As symptoms of atopic dermatitis improve, serum periostin level decreases to normal level^14^. We also found significantly elevated serum periostin in psoriatic patients compared to healthy individuals, but in contrast to patients with atopic dermatitis, interestingly, its level was the highest in systemically treated patients. Although analysis of serum periostin levels and age of psoriatic patients showed a tendentious correlation, we did not find any statistically significant association with other clinical characteristics of the patients. Similar to our observation in psoriasis, in rheumatoid arthritis, patients in remission had higher periostin serum levels compared to healthy individuals^24^, suggesting a relationship between serum periostin levels and improvement of the symptoms.

In atopic dermatitis, inflammation is characterized by enhanced fibroblast proliferation with an increased number of thickened collagen fibers, BM thickening, and elevated production of extracellular matrix proteins proteins, including periostin^15^. Similar tissue alterations are present in other Th2 pathway-mediated disorders such as asthma, in which serum periostin reflects inflammation activity, thus, it serves as a biomarker of acute flare of the disease^25^. As opposed to skin of atopic dermatitis we found periostin positivity in epidermal keratinocytes of lesional and even in non-lesional skin compared to healthy skin, at the same time, in the lesional psoriatic skin of untreated patients periostin distribution was decreased at the dermal–epidermal junction, which was confirmed by the mRNA expression and western blot analysis. Since the most prominent serum periostin expression was detected in the systemically treated psoriatic patients, we also analyzed periostin expression of previously-lesional healed skin. The previously-lesional skin showed an overall reduced periostin expression in the dermis, but basal keratinocytes showed the most prominent periostin positivity in the epidermis. Previously-lesional keratinocytes compared to healthy cells significantly overexpressed periostin in in vitro cultures, suggesting an activated state of the cells. In western blot analysis of periostin in cultured primary normal human keratinocytes and fibroblasts, we observed that the monomeric form of periostin was more characteristic for fibroblasts. Moreover, fibroblasts-derived monomeric periostin form was similar to what we detected in the whole tissue extracts by western blot analysis suggesting fibroblast contribution to the whole skin periostin content. Interleukin-4 and interleukin-13 can stimulate fibroblasts’ periostin production, and periostin expression is elevated in the lesional dermis of patients with atopic dermatitis, but expression changes in epidermal keratinocytes cannot be detected^16^. Interleukin-13 activates interleukin-24 in keratinocytes in a periostin-dependent way causing filaggrin downregulation, which results in an epithelial barrier dysfunction in atopic dermatitis^26^.

Several studies have described that both non-lesional and lesional psoriatic skin show similarities with wound repair processes^27^, and activation of keratinocytes is well-known during wound healing. A “pre-activated” state for hyperproliferation of keratinocytes has also been reported in the non-lesional skin^28^. It has been described that in mouse skin upon wounding, periostin was expressed by migrating keratinocytes^17^. Since BM abnormalities at the dermal–epidermal junction are characteristic alterations in psoriasis, and micro-wounds can be found along the BM, already in the non-lesional psoriatic skin, moreover wound-healing like changes are induced in keratinocytes^3–5,27^, we created the cultured salt-split model, to mimic skin with BM injuries. Increased periostin expression in keratinocytes in the cultured salt-split model indicates that induction of periostin depends on injuries localized at the dermal–epidermal junction. This wound-healing like process is also present in the non-lesional and lesional skin, and the healing process in the previously-lesional, resolved skin can remain switched on and be strengthened as a result of the therapy. However, further studies are needed to determine how long lasting these changes are in the healed skin. The elevated periostin level in the supernatant of our ex vivo and cultured salt-split wound models indicate that activation of basal keratinocytes leads to the release of cell-produced periostin, which could partially explain the elevated serum periostin levels we detected in the systemically treated psoriatic patients. Since previous animal studies have shown that periostin promotes arterial calcification and its deletion protects against atherosclerosis^21,22^, the increased serum periostin could play a role in the systemic inflammation described in psoriasis patients.

In atopic dermatitis the epidermal barrier injury is localized to the upper layer of keratinocytes, therefore we decided to use a tape-stripping type of injury to model the atopic dermatitis skin. In this model periostin expression in keratinocytes was not induced, indicating that a surface barrier epidermal damage does not induce periostin expression in keratinocytes. The abnormal BM structure of psoriatic skin can be sensed by integrins. Abnormal BM structures and injuries that affect the BM result in α5β1-integrin overexpression by keratinocytes^29^. We previously reported that β1-integrin and cartilage oligomeric matrix protein could interact in the psoriatic non-lesional skin due to the disrupted laminin layer^18^. β1-integrin blocking resulted in suppression of periostin expression in our scratch model indicating that β1-integrin can mediate periostin production upon wounding. PI3K/AKT is the main regulator of periostin expression and growth factors, transforming growth factor beta, and integrins can also activate periostin expression via this pathway^30^. Although, we did not examine the exact mechanism for how β1-integrin can induce periostin production, further experiments could reveal that in basal keratinocytes when basement membrane injury occurs, β1-integrin could influence through the PI3K/AKT pathway periostin expression. Taken together, abnormal BM-induced periostin expression of basal keratinocytes can be mediated by β1-integrin, which can act as a sensor of BM injuries.

**Figure 6:**
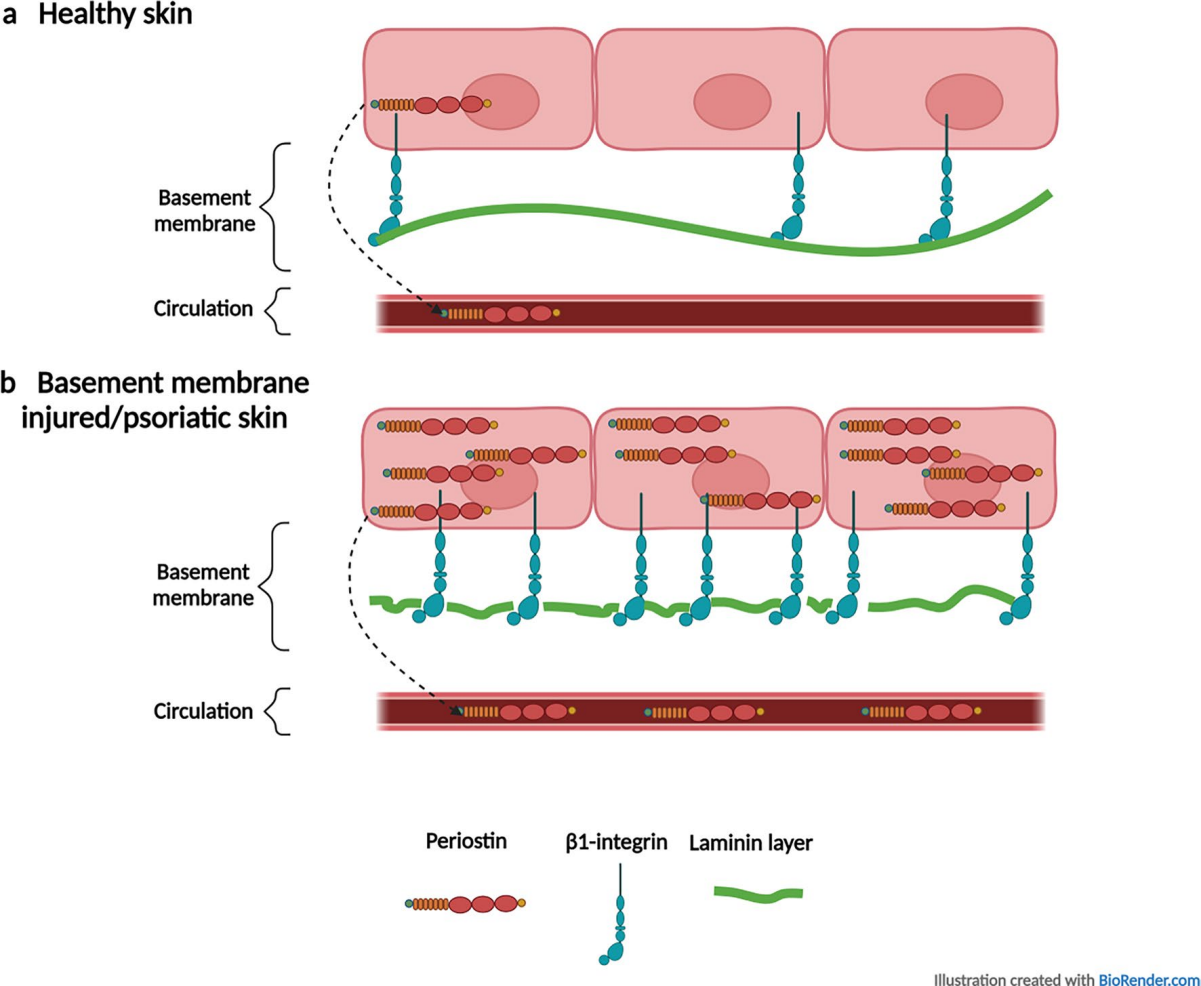
Abnormal basement membrane can induce periostin expression of basal keratinocytes, and β1-integrin can potentially act as a sensor of the basement membrane injuries and potentially contribute to the increased periostin expression. Schematic representation of (**a**) healthy and (**b**) basement membrane injured, psoriatic non-lesional, lesional, and previously-lesional skin. In healthy skin, the basement membrane is intact, the laminin layer is continuous and periostin and β1-integrin are normally expressed. In the basement membrane injured, cultured salt model, and psoriatic skin, the basement membrane is discontinuous, including the laminin layer, β1-integrin overexpressed for providing better stabilization for the cells, and periostin expression is also induced. The periostin production of these activated keratinocytes may also contribute to the elevated serum periostin levels in psoriatic patients. The illustration was created with BioRender.com.

Finally, this is the first study, which describes the elevated periostin expression in psoriatic keratinocytes, which could potentially contribute to the increased serum periostin detected in this disease. In contrast to lesional skin of atopic dermatitis, where Th2-type cytokines stimulate fibroblasts to increase periostin production, in psoriatic skin basal keratinocytes play a key role in enhanced periostin production. Our results suggest that basal keratinocytes are in an activated state in the non-lesional, lesional, and even more so in the previously-lesional psoriatic epidermis and they show a stable wound healing-like phenotype with the overexpression of periostin reflecting the abnormal BM. β1-integrin, also overexpressed in the cells, contributes to enhanced periostin production (Fig. 6). Our results also demonstrate how tissue resident cells could be differentially activated by distinct spatial changes in tissue. The abnormal BM-induced wound healing as a potential compensatory mechanism is initiated already in the non-lesional skin, it is present in the lesion, and it can be amplified as a result of the therapy and remain active in the healed skin.

## Materials and methods

### Blood, skin samples and ethics

In this study, we recruited patients with chronic plaque-type psoriasis and their initial Psoriasis Area Severity Index (PASI) scores were determined. Blood serum samples were collected from 105 patients in total with chronic plaque-type psoriasis and 49 healthy volunteers. The characteristics of Psoriatic patients are listed in Table 1. Untreated patients (n = 41) did not receive topical therapies for 4 weeks and systemic treatments for 8 weeks before blood collection. Treated patients (n = 64) received either different types of biological therapies (TNF-α inhibitors, anti-IL-12- and IL-23p40 antibody, anti-IL-17 antibody and anti-IL-23p19 antibody), or immunosuppressants (methotrexate, steroid, acitretin). Initial PASI values were determined.

**Table 1:** Clinical characteristics of psoriatic (PS) patients and healthy (H) individuals.

Patients data	H volunteers	Untreated PS patients	Treated PS patients
Patients number	49	41	64
Initial median PASI	–	16.6	16.6
Initial average PASI	–	20.5	18.3
Initial PASI range	–	5.0–61.2	3.3–37.5
Genders	31 males	34 males	40 males
	18 females	7 females	24 females
Median age	48	56	58
Biological therapies	–	–	43
Immunosuppressants			21
Types of biological therapies			
TNF-α inhibitors	–	–	15
anti-IL-12- and IL-23p40 antibody	–	–	17
anti-IL-17 antibody	–	–	9
anti-IL-23p19 antibody	–	–	2
Types of immunosuppressants			
Methotrexate	–	–	19
Steroid	–	–	1
Acitretin			1

Skin punch biopsies (dia = 6 mm) were collected from untreated psoriatic patients from lesional (n = 4) and non-lesional (n = 4, at least 6 cm from the lesion) skin areas and from healthy individuals (n = 4). Punch biopsies were also collected from systemically treated patients from their previously-lesional, healed (n = 4) skin areas. Following the rules of the Helsinki Declaration, all donors provided written informed consent before sample collection. The protocols for this study were approved by the Regional and Institutional Research Ethics Committee (HCEMM-001, 10/2020, 4702, 20 January 2020; PSO-VA0223-001, 65/2018, 4236, 19 March 2018, Szeged, Hungary; PSO-CELL-01, 90/2021, 4969, 26 April 2021, Szeged, Hungary; PSO-EDAFN-002, 34/2015, 3517, 23 February 2015, Szeged, Hungary).

### Immunofluorescence labeling

Frozen, 4% paraformaldehyde fixed and 0.25% TritonX-100 (Sigma Aldrich, Saint Louis, Missouri, USA) permeabilized 6 µm skin sections and were blocked with 3% normal goat serum and 1% bovine serum albumin containing (both Sigma Aldrich, Saint Louis, Missouri, USA) Tris-buffered saline. For immunolabeling mouse anti-human periostin (1:125, #sc‐398631, Santa Cruz Biotechnology), and β1-integrin (1:100, #ab30394, Abcam, Cambridge, UK) were used overnight followed by Alexa Fluor 647 conjugated goat anti-mouse IgG (Life Technologies, Carlsbad, California, USA). As isotype control mouse IgG1κ (#400102, BioLegend, San Diego, California, USA) was used, 4ʹ,6-diamidino-2-phenylindole (DAPI, 1:100, Sigma Aldrich) labeled the nuclei. Visualization, image processing and fluorescence quantification were performed by Zeiss Axio Imager Z1 microscope, ZEN 2012 Microscope Imaging software (Carl Zeiss AG, Oberkochen, Germany) and Fiji software (ImageJ, Wisconsin, USA).

### Determination of periostin in the serum

Periostin levels in the serum were measured by sandwich enzyme-linked immunosorbent assay (ELISA, R&D Systems, Minneapolis, Minnesota, USA) kits according to manufacturer’s instruction.

### Tape-stripping, ex vivo human skin wound healing and cultured salt-split models

For tape-stripping model (n = 3), biopsies were tape-striped by adhesive tape 10 times. For the cutting-type ex vivo wound healing models (n = 5), skin pieces were cut out of healthy skin, shaped into approximately 1 cm diameter pieces, then wounded by a 4 mm punch biopsy scalpel (Steele Supply Company, St. Joseph, MI, USA). For the cultured salt-split (n = 5), 6 mm punch biopsies were incubated in 1 M NaCl (Sigma-Aldrich, Saint Louis, Missouri, USA) for 5 h at 4 °C. All skin samples were then cultured for either 24 or 72 h at an air–liquid interface in transwell cell culture inserts (Corning Inc., Corning, NY, USA) in 10% fetal bovine serum (FBS, EuroClone, Pero, Italy) containing DMEM F12 (Lonza Group, Basel, Switzerland) media supplemented with 1% antibiotic/antimycotic solution (Sigma-Aldrich, Saint Louis, Missouri, USA). Samples were embedded in cryogenic solution (Thermo-Fischer Scientific, Waltham, Massachusetts, USA) for stainings, and supernatants (n = 3) were collected at 0, 24 h from ex vivo and 0, 24 and 72 h from cultured salt-split models.

### In vitro scratch assay

Primary normal human keratinocytes were plated onto a 6-well plate at a density of 5 10^5^, then 1 µg/ml β1-integrin blocking antibody (#ab30394, Abcam, Cambridge, UK, and #303004, BioLegend, San Diego, California, USA) was added 5 h post-seeding. After 24 h, 100% confluent cultures were scratched and cultured for 24 h and cells were harvested for western blot analysis. The wound closure was monitored with a Zeiss Axiolab Vert.A1 microscope (Carl Zeiss AG, Oberkochen, Germany).

### Further methods

More detailed information on the materials and methods is presented in the Supplementary Data.

### Supplementary Information

Supplementary Information 1.

Supplementary Information 2.

## Data Availability

The datasets used and analyzed during the current study are available from the corresponding author on reasonable request.
