# Supplementary material for: Abnormal basement membrane results in increased keratinocyte-derived periostin expression in psoriasis similar to wound healing
Source: Sci Rep. 2023 Sep 29;13:16386. doi: 10.1038/s41598-023-43396-0 (PMC10541889; doi:10.1038/s41598-023-43396-0)
Supplement: Supplementary file 1 — Supplementary Information 1. [file 41598_2023_43396_MOESM1_ESM.docx]

**Supplementary Data**

| Patients data | H volunteers | | Untreated PS patients | | Treated PS patients |
| --- | --- | --- | --- | --- | --- |
| **Patients number** | 49 | | 41 | | 64 |
| **Initial median PASI** | - | | 16.6 | | 16.6 |
| **Initial average PASI** | - | | 20.5 | | 18.3 |
| **Initial PASI range** | - | | 5.0-61.2 | | 3.3-37.5 |
| **Genders** | 31 males  18 females | | 34 males  7 females | | 40 males  24 females |
| **Median age** | 48 | | 56 | | 58 |
| **Biological therapies** | - | | - | | 43 |
| **Immunosuppressants** |  | |  | | 21 |
|  | **Types of biological therapies** | | | | |
| **TNF-α inhibitors** | **-** | **-** | | 15 | |
| **anti-IL-12- and IL-23p40 antibody** | **-** | **-** | | 17 | |
| **anti-IL-17 antibody** | **-** | **-** | | 9 | |
| **anti-IL-23p19 antibody** | **-** | **-** | | 2 | |
|  | **Types of immunosuppressants** | | | | |
| **Methotrexate** | - | | - | | 19 |
| **Steroid** | - | | - | | 1 |
| **Acitretin** |  | |  | | 1 |

**Table 1. Clinical characteristics of psoriatic (PS) patients and healthy (H) individuals**

**Supplementary materials and methods**

**Cell cultures**

Healthy and previously-lesional psoriatic human skin biopsies were washed in Salsol A (Human Rt, Gödöllő, Hungary) containing 2% antibiotic/antimycotic solution (Sigma-Aldrich, Saint Louis, Missouri, USA). Punch biopsies were cut into small pieces and incubated in Dispase II (Roche Diagnostics, Manheim, Germany) solution overnight, then the epidermis and dermis were separated. Primary human keratinocytes were isolated from the epidermis after incubation in trypsin-EDTA solution (Sigma Aldrich, Saint Louis, Missouri, USA) for 5 minutes at 37 °C to achieve an epidermal cell suspension. Human fibroblasts were obtained from the dermis after incubation in Digestion Mix (Collagenase, Hyaluronidase, and Deoxyribonuclease, Sigma Aldrich, Saint Louis, Missouri, USA) for 2h at 37°C. The cell suspensions were filtered through 100 µm strainers (BD Falcon, San Jose, CA, USA) and pelleted by centrifugation. Primary keratinocytes were cultured in epidermal growth factor and bovine pituitary extract containing serum-free media (Gibco Keratinocyte SFM Kit; Life Technologies, Copenhagen, Denmark), while fibroblasts were grown in low glucose DMEM (Lonza Group, Basel, Switzerland) media containing 5% FBS (EuroClone, Pero, Italy). Both types of media were supplemented with 1% antibiotic/antimycotic solution (Sigma Aldrich, Saint Louis, Missouri, USA) and 1% ʟ-glutamine (PAA Laboratories GmBH, Pasching, Austria). Cells were cultured in 75 cm^2^ cell-culture flasks at 37 °C and 5% CO^2^ in humidified conditions. Cell culture media were changed every 2–4 days and cells were passaged at 80% confluence. Keratinocytes were used in the third passage, fibroblasts were used in the fifth passage at 80% confluency for the experiments.

**Gene expression data analysis**

To analyze periostin gene expression, GEO Profile Database (GDS4602 datasets, ID:100674764) was used, which stores publicly available microarray data from total RNA content derived from healthy (n=64), psoriatic lesional (n=58) and non-lesional (n=58) whole skin punch biopsies.

**Western blot**

Punch biopsies were cut and incubated in a 6 M guanidine hydrochloride (Sigma-Aldrich, Saint Louis, Missouri, USA) solution. The supernatant was collected and ethanol-based precipitation was performed, then the pellet was dissolved in 3 M urea (Sigma-Aldrich, Saint Louis, Missouri, USA). Fibroblast and keratinocyte cultures were collected in phosphate-buffered saline and then lysed in 20 mM 4-(2-hydroxyethyl)-1-piperazineethanesulfonic acid (HEPES), 150 mM potassium chloride (KCl), 1 mM magnesium chloride (MgCl^2^), 1 mM dithiothreitol (DTT), 10% glycerol, 0.1% NP-40 (all from Sigma-Aldrich, Saint Louis, Missouri, USA) and 5% TritonX-100 (Sigma-Aldrich, Saint Louis, Missouri, USA), supplemented with 1% protease inhibitor cocktail, 1% phenylmethylsulfonyl fluoride (PMSF) and 5% of 10% sodium dodecyl sulfate (SDS) (all from Sigma-Aldrich, Saint Louis, Missouri, USA). Cell lysates and supernatants of *ex vivo* wound healing and cultured salt split models were boiled for 5 minutes with 4X loading buffer (Lonza Group, Basel, Switzerland), tissue extracts were boiled for 10 minutes with 4X loading buffer (Lonza Group, Basel, Switzerland) supplemented with β-mercaptoethanol (Sigma-Aldrich, Saint Louis, Missouri, USA) then all extracts were electrophoresed on 4–20% Mini-PROTEAN®TGX™ Precast Gels and transferred to nitrocellulose membranes (all from Bio-Rad, Hercules, California, USA), finally stained with PonceauS (Thermo Fischer Scientific, Waltham, Massachusetts, USA). Membranes were blocked with 5% non-fat milk powder containing Tris-buffered saline either supplemented with or without 1% bovine serum albumin, then incubated overnight with mouse anti-human periostin (1:500, #sc‐398631, Santa Cruz Biotechnology, Dallas, Texas, USA), rabbit anti-human periostin (1:1000, #NBP1-30042, Novus Bio, Centennial, CO, USA), rabbit anti-human actin (1:2000, #A2066, Sigma-Aldrich, Saint Louis, Missouri, USA) and mouse anti-human GAPDH (1:1000, #G8795, Sigma-Aldrich, Saint Louis, Missouri, USA) either with or without membrane stripping with 100 mM glycine (Sigma-Aldrich, Saint Louis, Missouri, USA) solution. Detection was performed using horseradish peroxidase‐conjugated secondary antibodies (1:2000, Southern Biotech, Birmingham, Alabama, USA) and bands were visualized by an enhanced chemiluminescent system (Bio-Rad, Hercules, California, USA) with a LI-COR C-DiGit Blot Scanner (LI-COR, Lincoln, Nebraska, USA).

**Hematoxylin eosin staining**

Hematoxylin-eosin (H&E, Leica Biosystems, Wetzlar, Germany) staining was performed on tape-stripping, *ex vivo* wound healing, and cultured salt split models according to the manufacturer’s instructions in a Leica ST5020 Multistainer device (Leica Biosystems, Wetzlar, Germany).

**Statistical analysis**

All data were normalized to control and were presented as mean ± standard error of the mean. Comparisons between two groups were tested for statistical significance by either one- or two-tailed two-sample *t*-test, for more than two groups Kruskal-Wallis or one-way ANOVA tests were used followed by Pairwise Wilcoxon test or Tukey’s post hoc test according to the figure legends. Correlations were determined by Spearman’s rank test. ****P* <0.0001, ***P* <0.01 or **P* <0.05 were considered statistically significant. Data analysis and illustration were performed either using R-Studio software (version 4.1.3 R-Studio, Boston, USA) or Prism-GraphPad 8 software (Graphpad Software Inc. version 8.0.2, San Diego, California, USA).

**Supplementary figures**

**
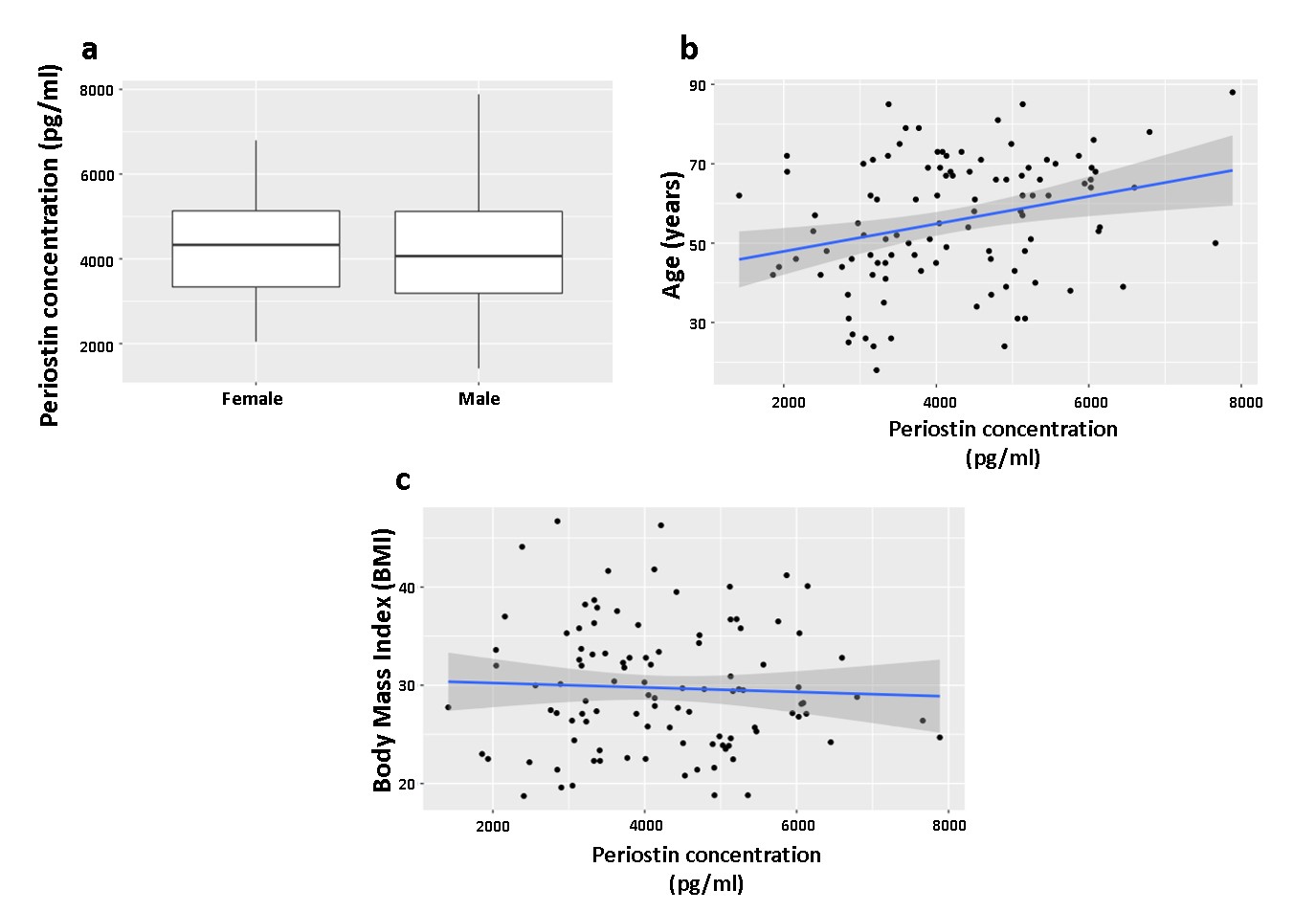
Supplementary Figure S1. Elevated serum periostin levels are independent of gender, age, and BMI in psoriatic patients.**

(a) Serum periostin levels in female and male psoriatic patients. *P* values are calculated by two-sided two-sample *t*-tests. (b) Correlation between serum periostin and age (n=105, r=0.291, *P*=0.003). (c) Correlation between serum periostin levels and Body Mass Index (BMI) values (n=105, r=-0.023, *P*=0.891). Correlations were determined by Spearman’s rank correlation test.


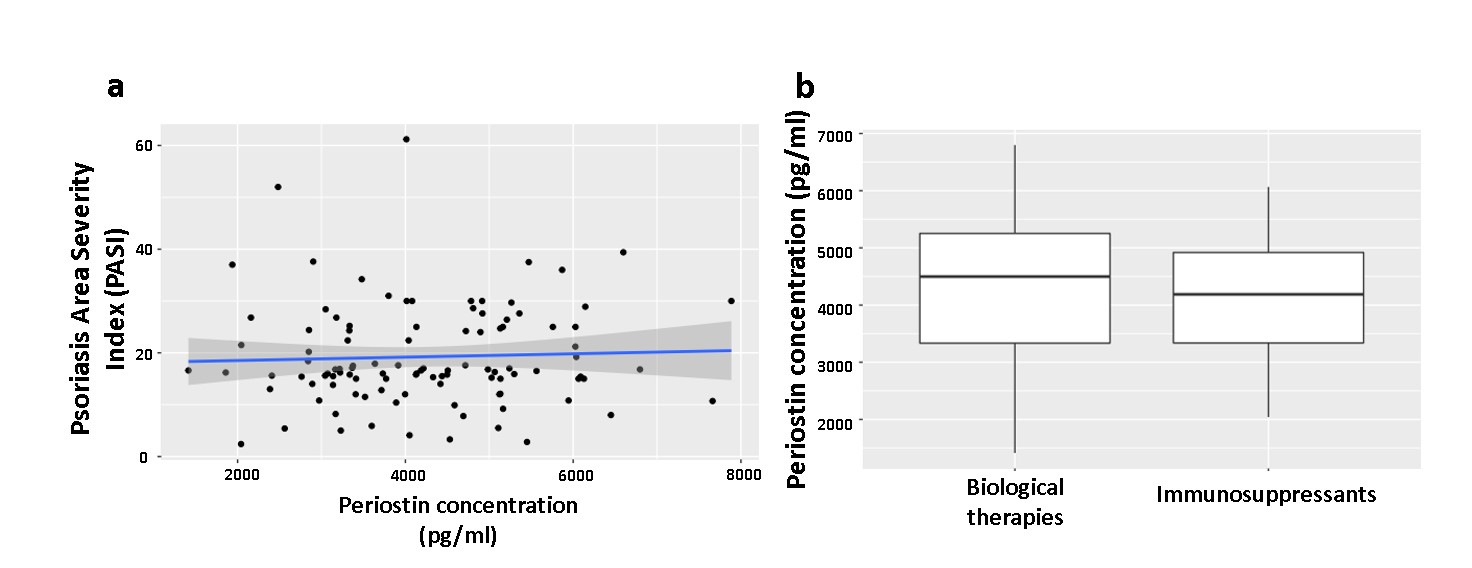
**Supplementary Figure S2.: Elevated serum periostin levels are independent of disease severity and the type of systemic treatment in psoriatic patients.**

(a) The correlation between serum periostin levels and Psoriasis Area and Severity Index (PASI) values (n=105, r=0.057, *P*=0.563). (b) The correlation was determined by Spearman’s rank correlation test. Comparison of serum periostin levels in psoriatic patients treated with biological therapies and immunosuppressants. *P* values are calculated by two-sided two-sample *t*-tests.

**
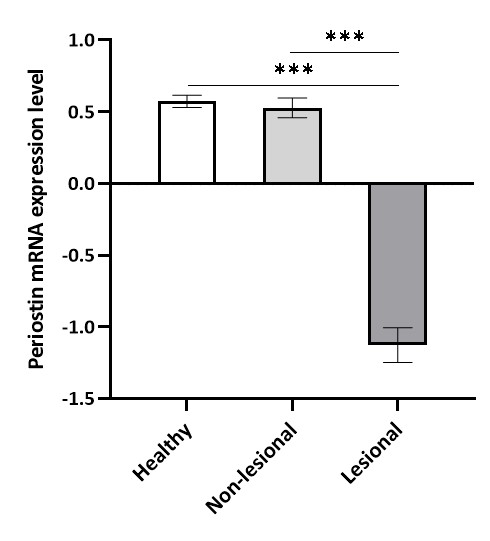
Supplementary Figure S3.: Periostin mRNA expression is decreased in the lesional psoriatic skin.**

Periostin expression profile from the publicly available GEO Profile data was analyzed and compared with one-way ANOVA test followed by Tukey’s posthoc test. The graph shows mean±SD of healthy (n=64), non-lesional (n=58), and lesional (n=58) skin. ***:*P* <0.0001 was considered statistically significant.

**
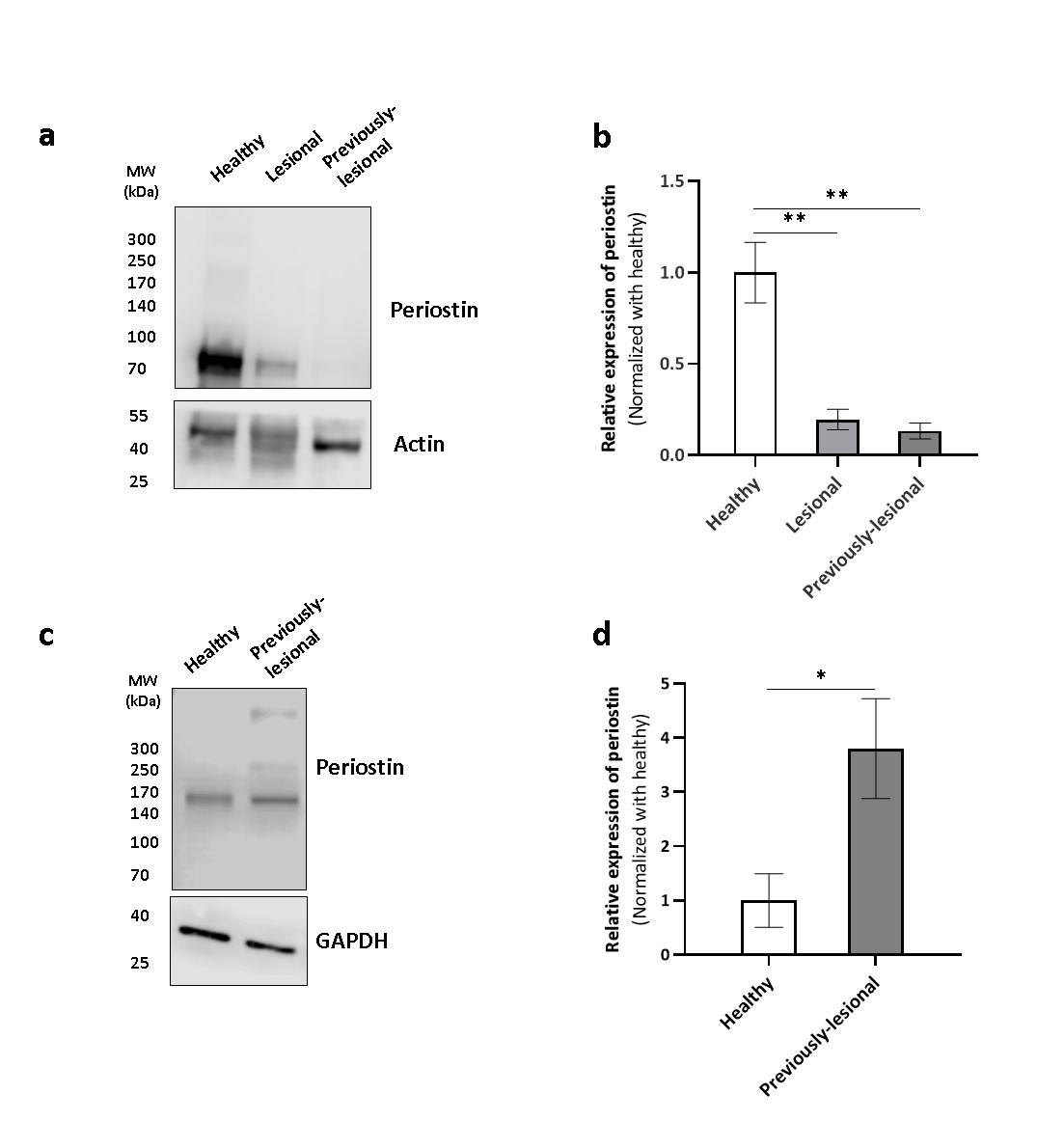
Supplementary Figure S4.:** **Periostin is decreased in the lesional and previously-lesional psoriatic whole skin, but increased in cultured, previously-lesional psoriatic keratinocytes.**

(a) Western blot analysis of periostin in healthy, lesional, and previously-lesional tissue extracts and in previously-lesional and healthy primary keratinocyte lysates. Representative data from western blot analysis from 3 independent donors are shown. (b) Periostin bands were quantified by Image Studio software (LI-COR Biosciences, Lincoln, Nebraska, USA). Data were normalized to actin. The graph shows mean±SEM (n=3) of L, PL vs. H. **:*P* <0.01 calculated by one-way ANOVA, followed by Tukey’s posthoc test. (c, d) Previously-lesional and healthy keratinocytes’ periostin expressions were analyzed by western blot. GAPDH was used as loading control. Data were normalized to GAPDH. The graph shows the mean±SEM (n=3) of PL vs. H cultured keratinocytes. **P* <0.05, determined by one-tailed two-sample *t*-test.


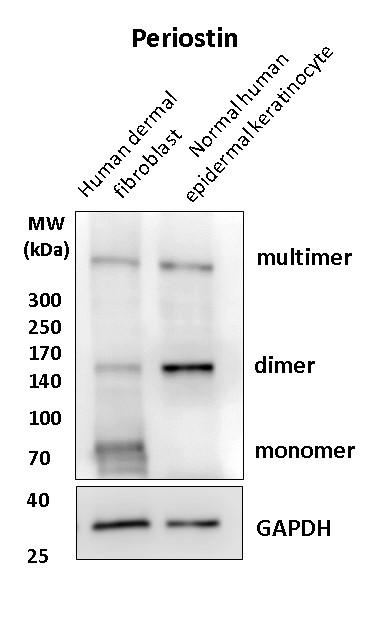
**Supplementary Figure S5.:** **Primary human keratinocytes and fibroblasts show characteristic bands of periostin.**

Western blot analysis of periostin in cultured normal human epidermal keratinocyte and human dermal fibroblast lysates. Representative image from western blot analysis from 4 independent donors are shown. GAPDH was used as loading control.
