# Supplementary material for: Abnormal basement membrane results in increased keratinocyte-derived periostin expression in psoriasis similar to wound healing
Source: Sci Rep. 2023 Sep 29;13:16386. doi: 10.1038/s41598-023-43396-0 (PMC10541889; doi:10.1038/s41598-023-43396-0)

Original Western blot images

Figure 3d

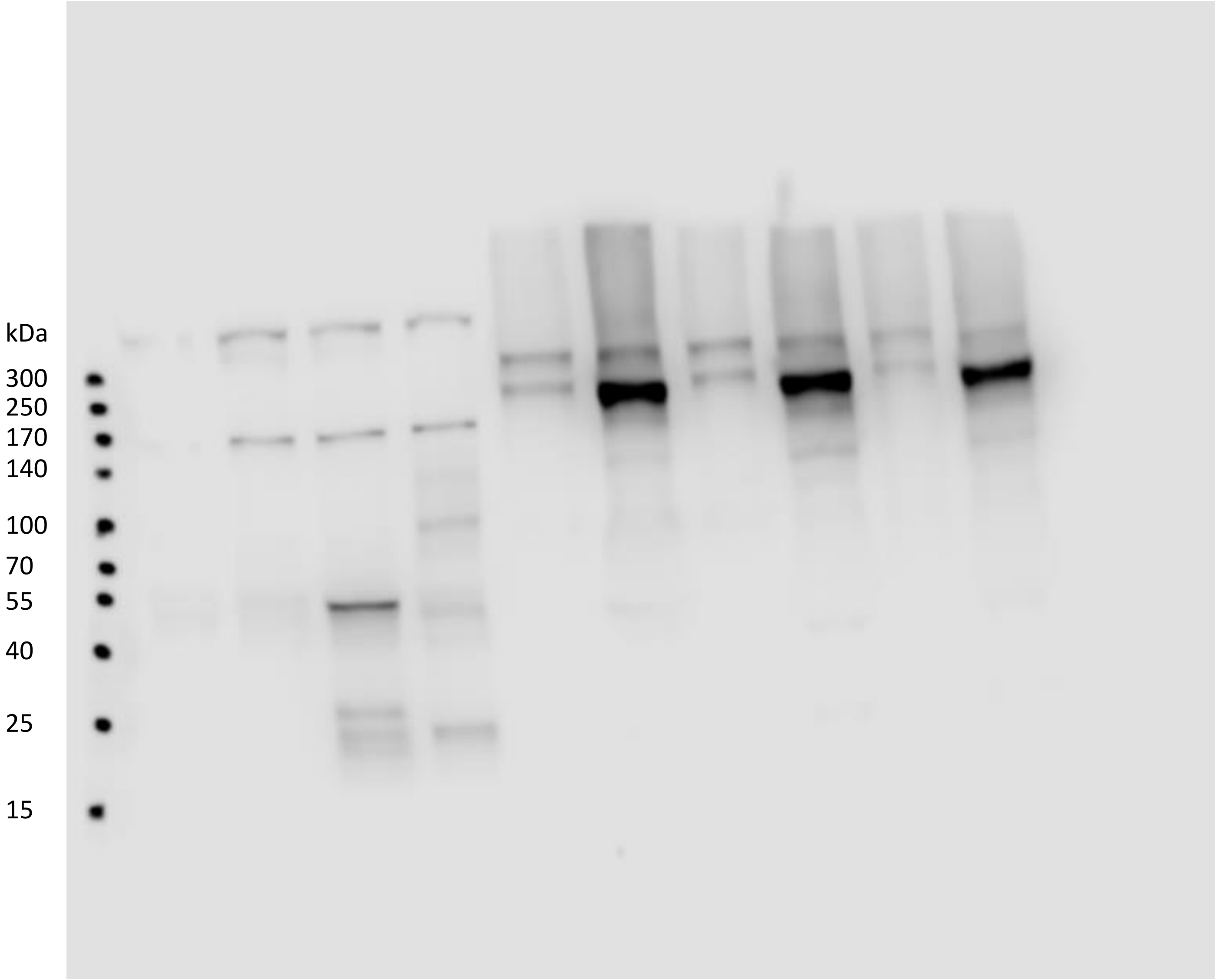

Original Western blot images

Figure 3e

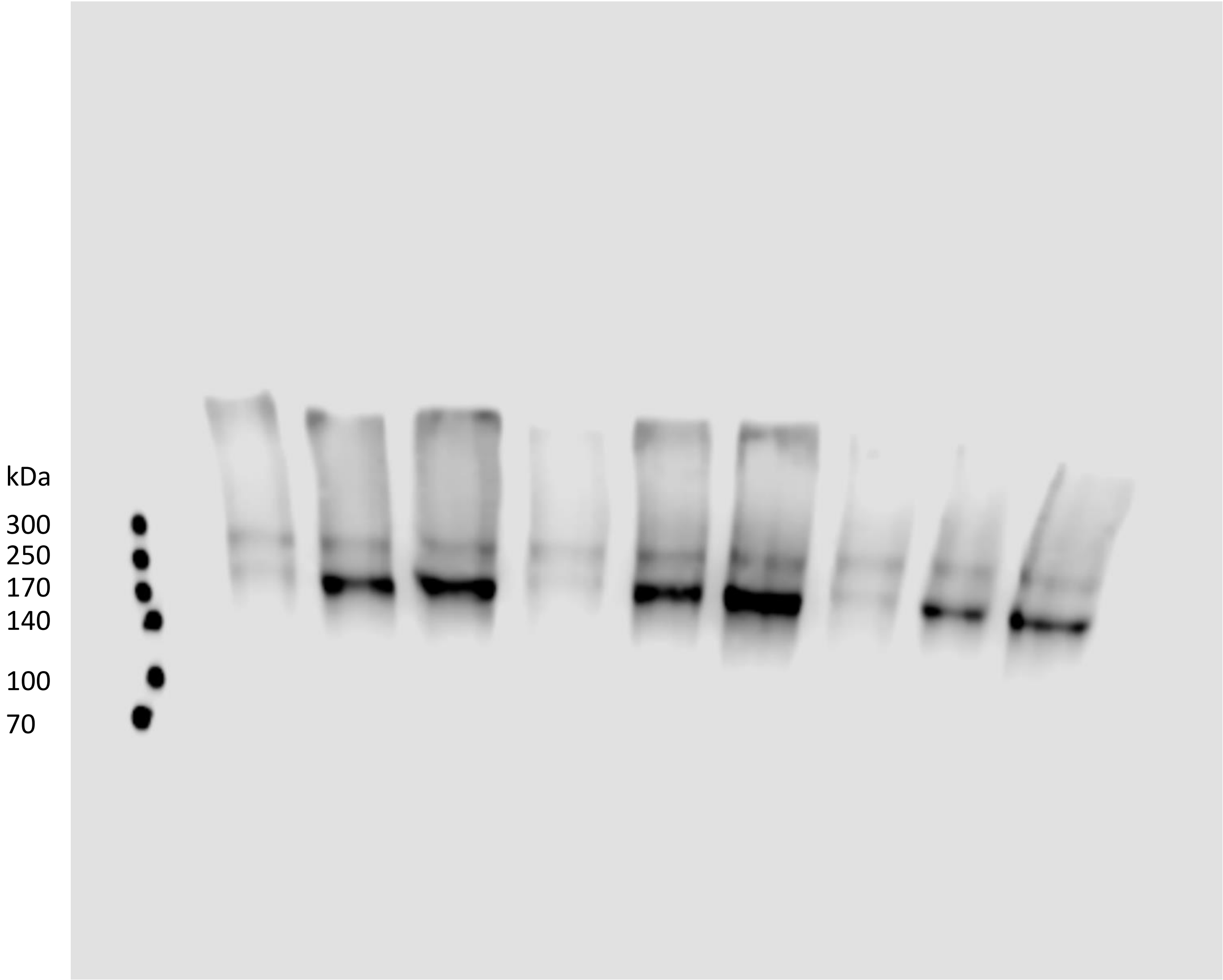

Original Western blot images

Figure 5b

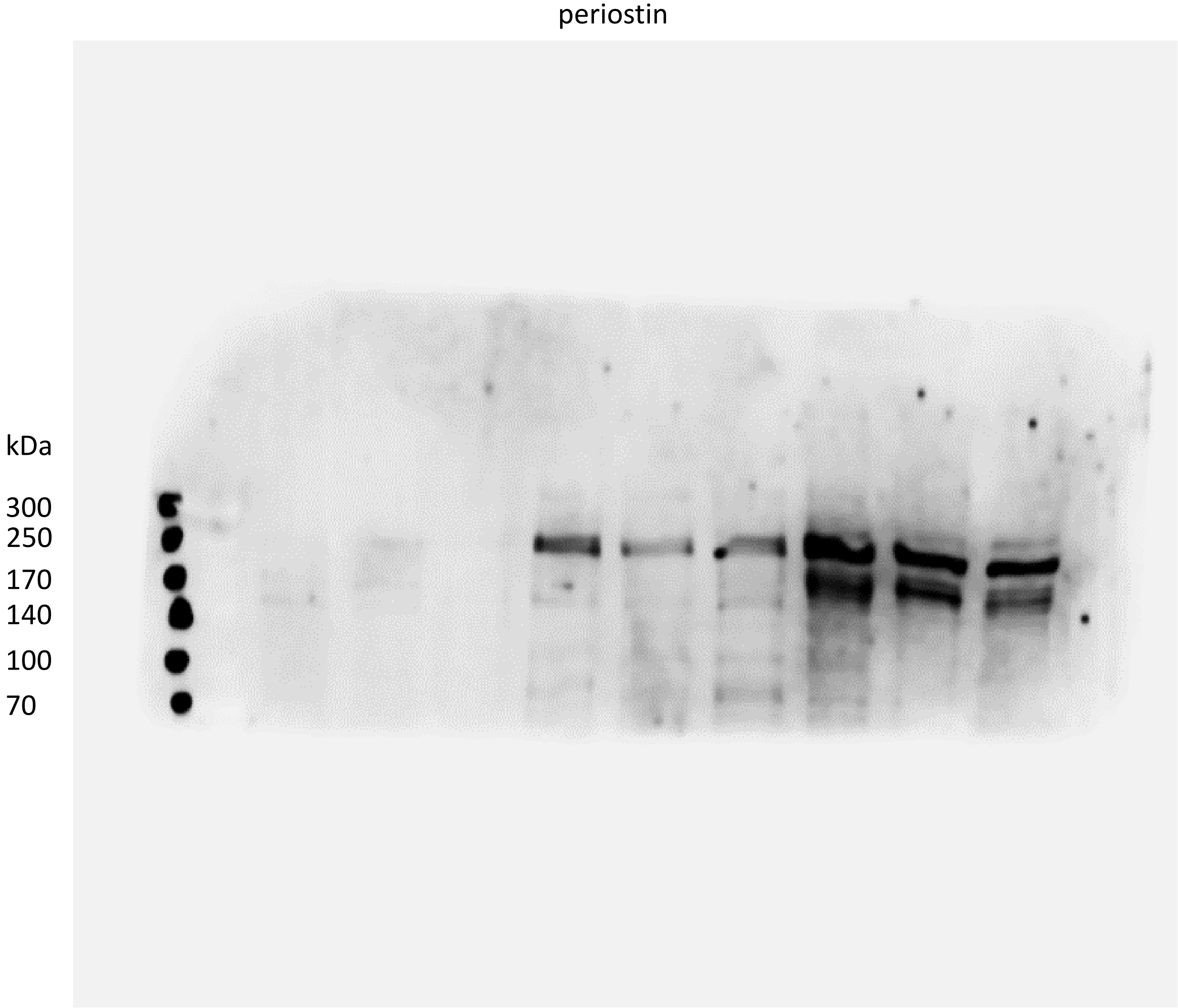

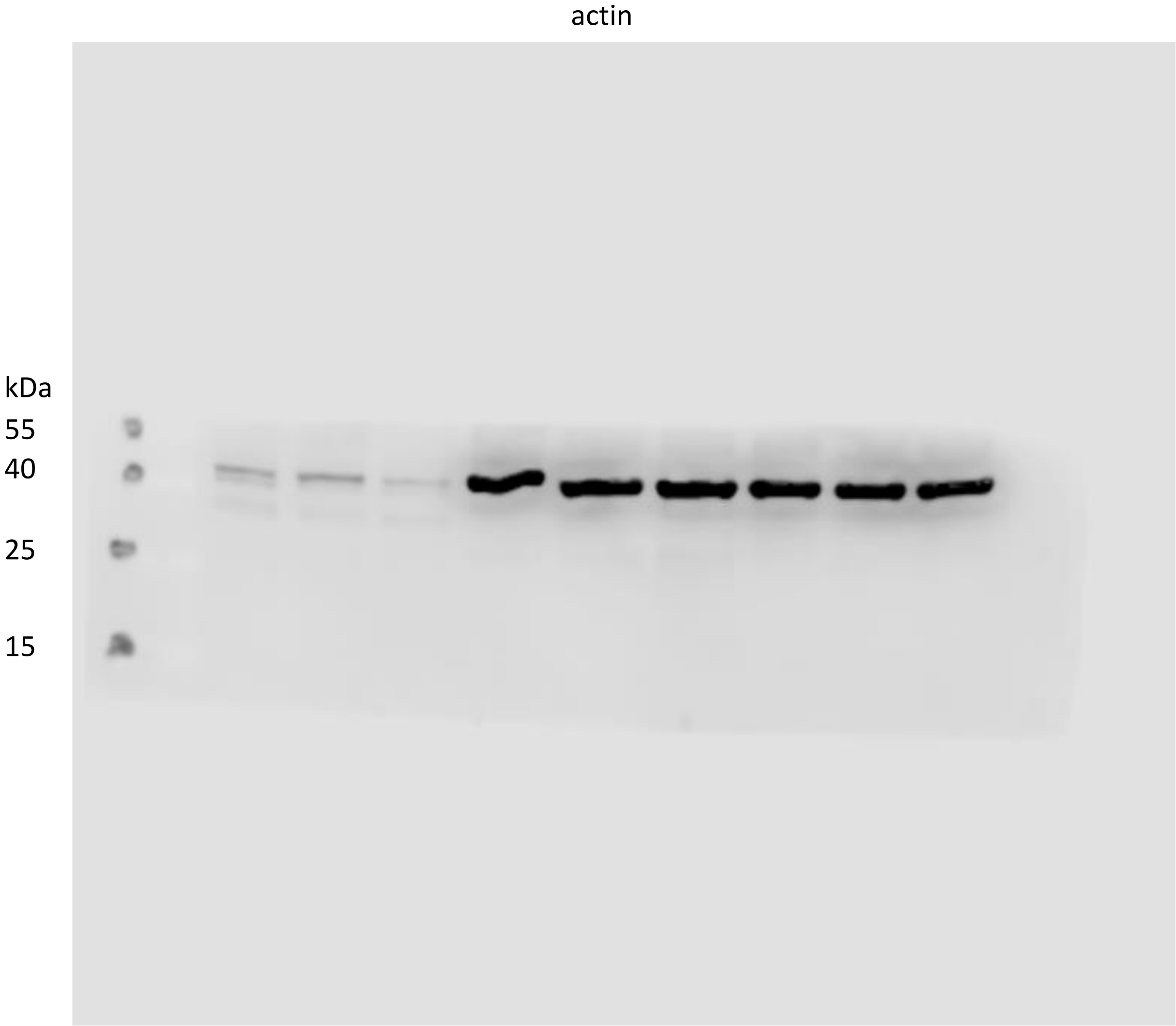

Original Western blot images

Figure S4a

periostin

kDa
300
250
170
140
100
70
55
40
25

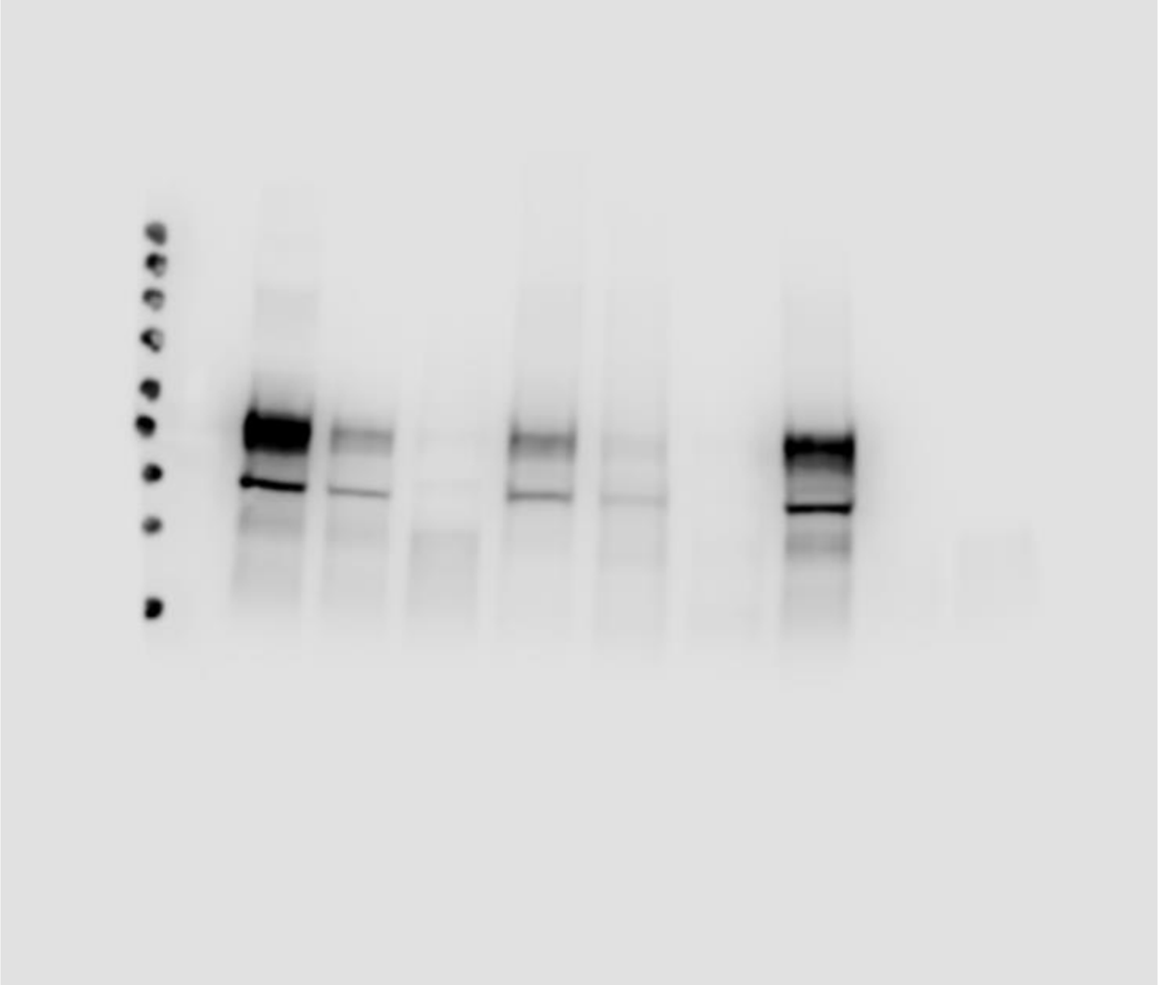

actin

kDa
55
40
25

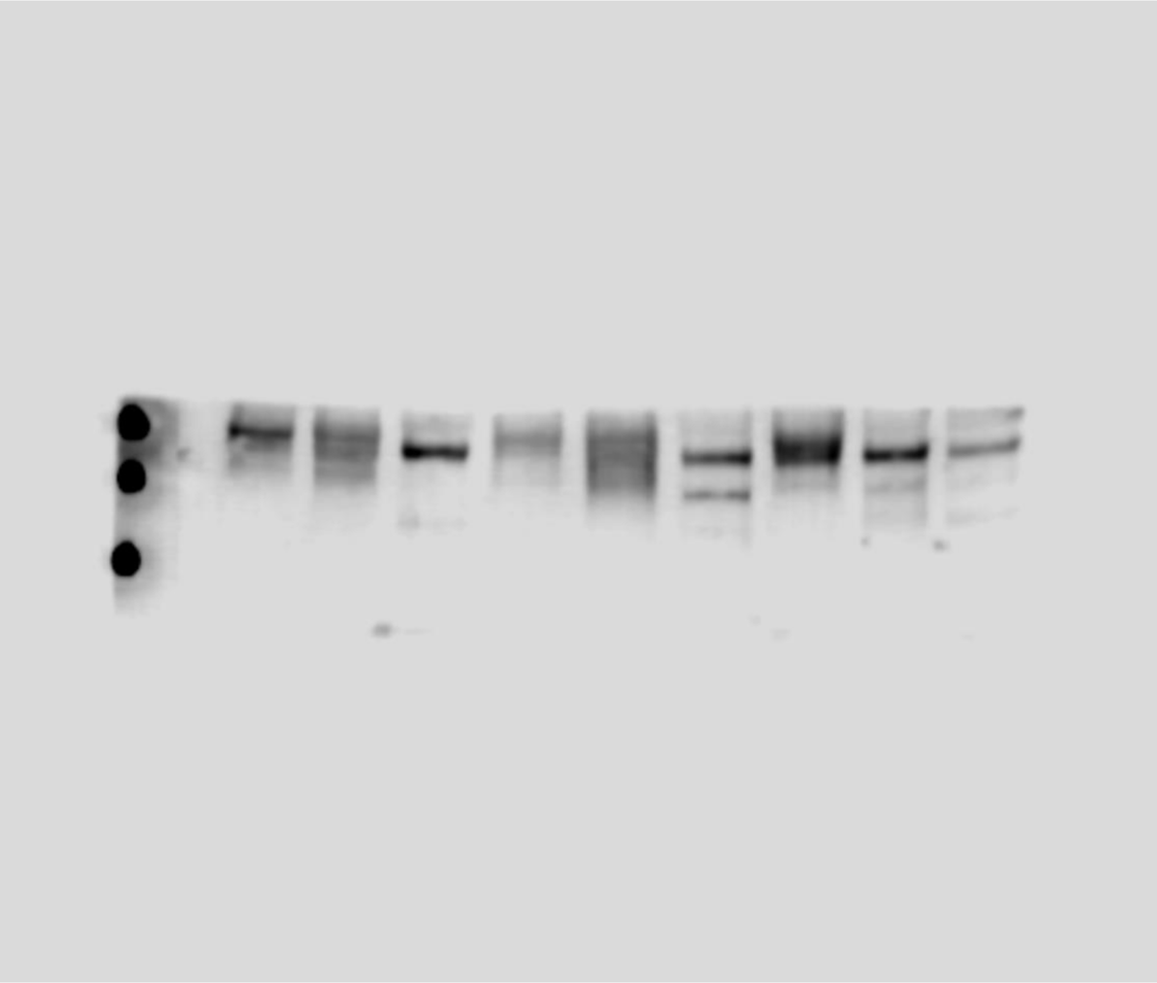

Original Western blot images

Figure S4c

periostin

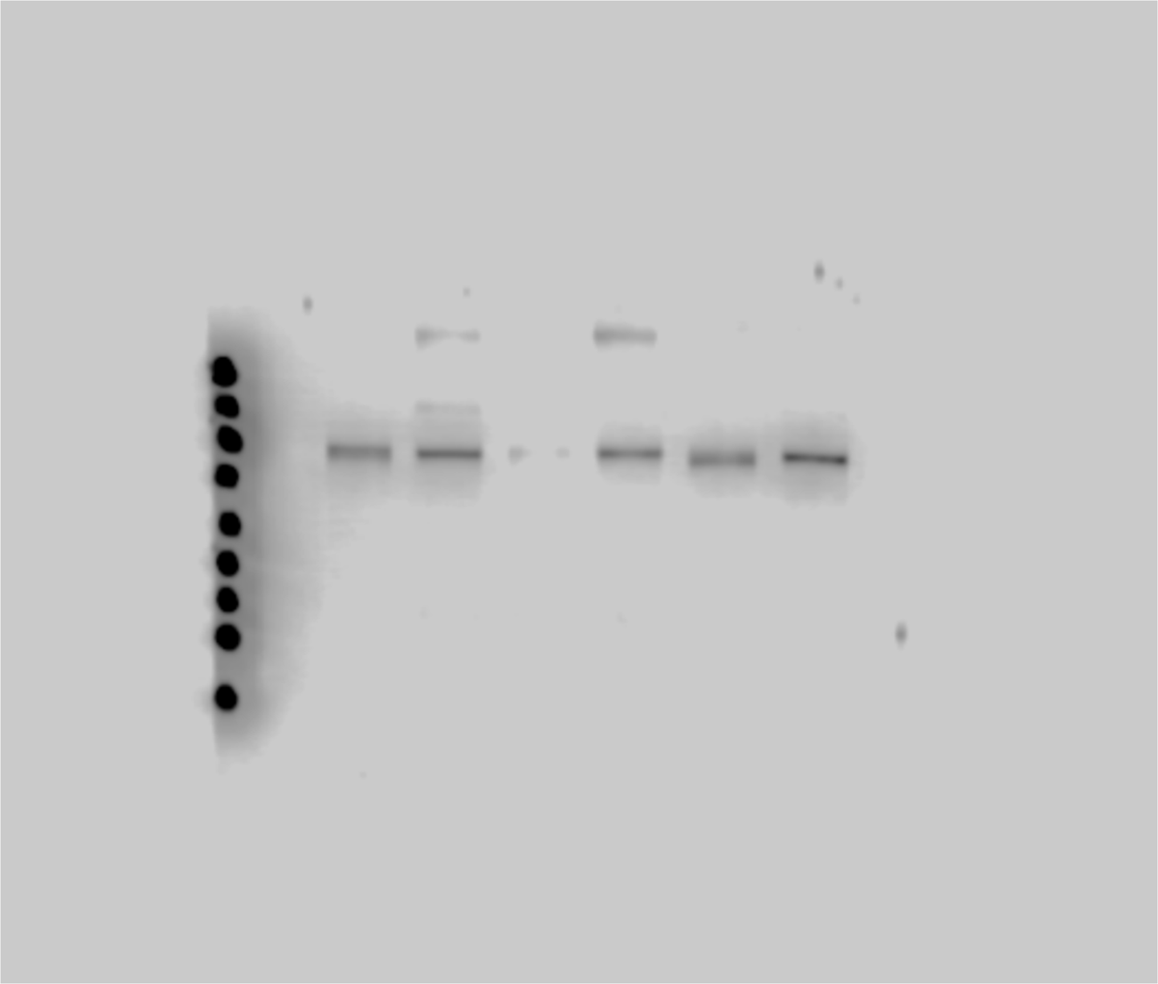

GAPDH

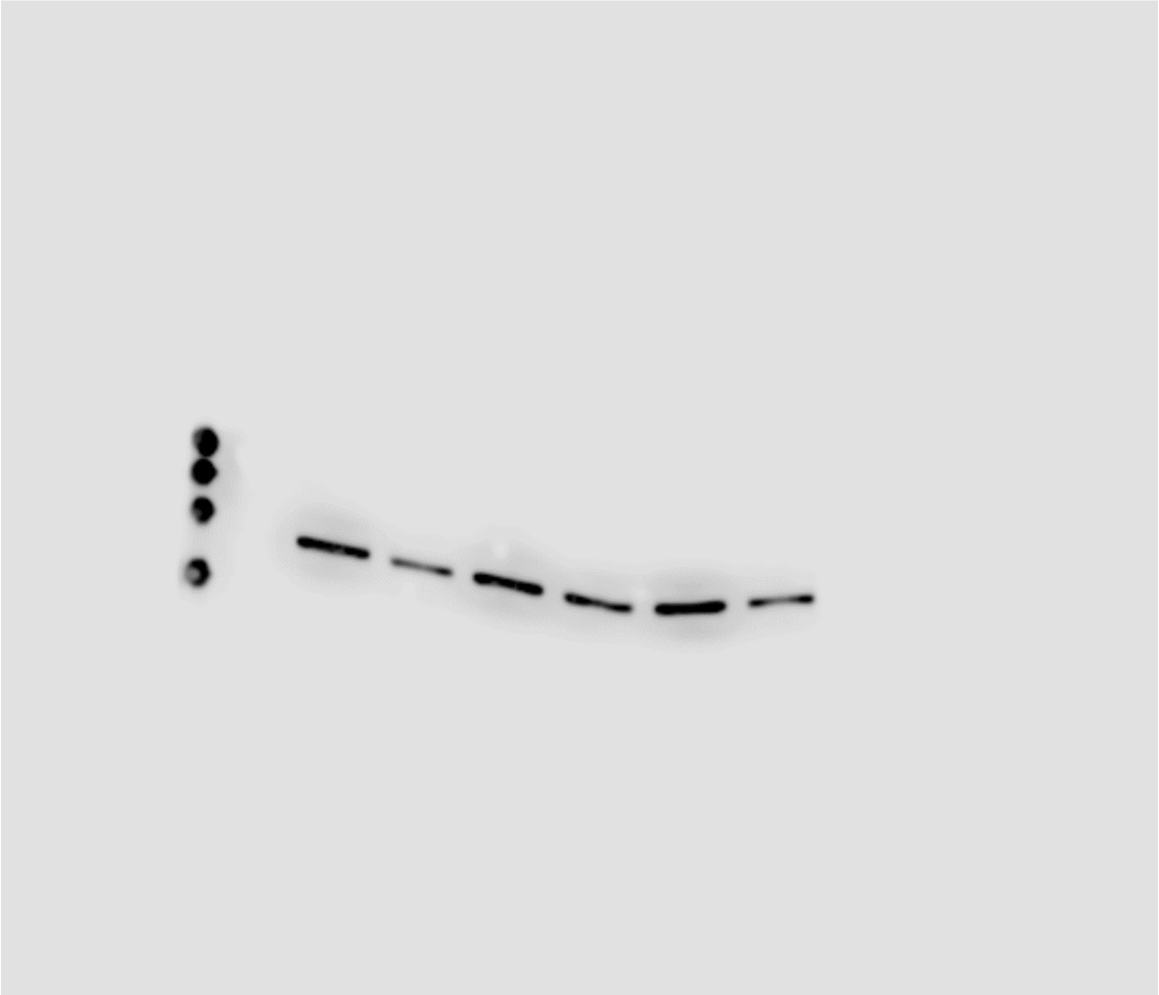

Original Western blot images

Figure S5

periostin

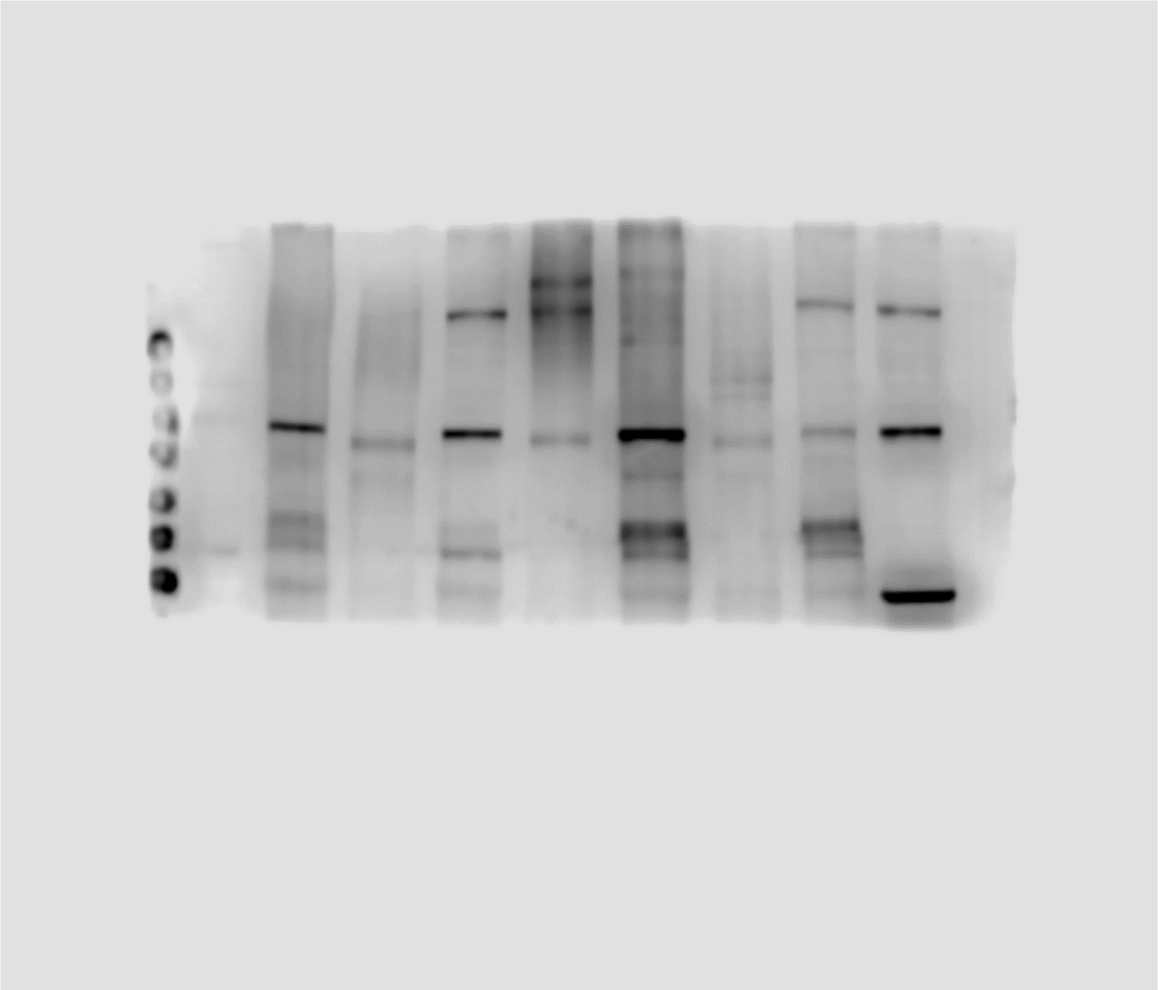

GAPDH

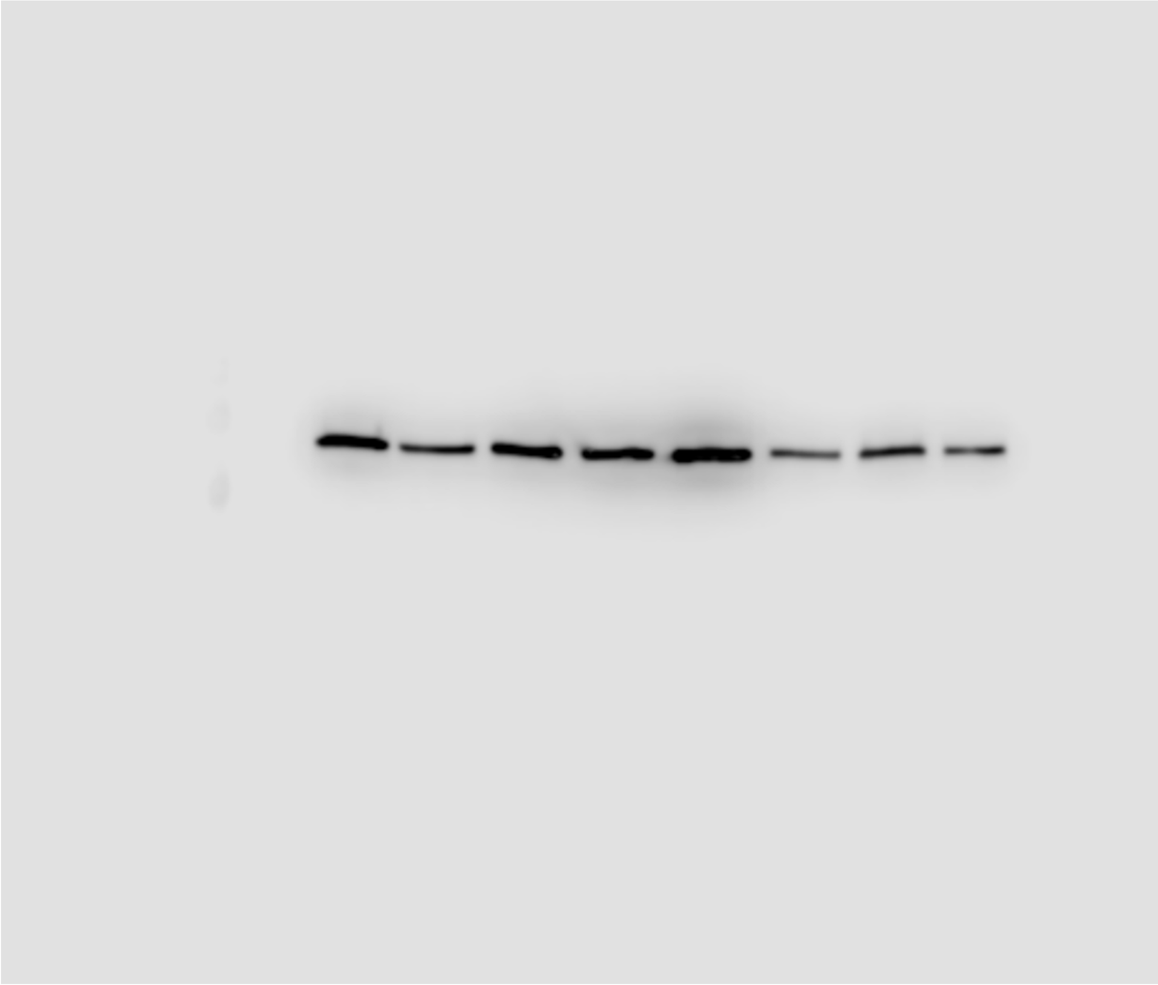

Supplement: Supplementary file 2 — Supplementary Information 2. [file 41598_2023_43396_MOESM2_ESM.pdf]
